# Whole Genome, Whole Population Sequencing Reveals That Loss of Signaling Networks Is the Major Adaptive Strategy in a Constant Environment

**DOI:** 10.1371/journal.pgen.1003972

**Published:** 2013-11-21

**Authors:** Daniel J. Kvitek, Gavin Sherlock

**Affiliations:** Department of Genetics, Stanford University, Stanford, California, United States of America; University of Michigan, United States of America

## Abstract

Molecular signaling networks are ubiquitous across life and likely evolved to allow organisms to sense and respond to environmental change in dynamic environments. Few examples exist regarding the dispensability of signaling networks, and it remains unclear whether they are an essential feature of a highly adapted biological system. Here, we show that signaling network function carries a fitness cost in yeast evolving in a constant environment. We performed whole-genome, whole-population Illumina sequencing on replicate evolution experiments and find the major theme of adaptive evolution in a constant environment is the disruption of signaling networks responsible for regulating the response to environmental perturbations. Over half of all identified mutations occurred in three major signaling networks that regulate growth control: glucose signaling, Ras/cAMP/PKA and HOG. This results in a loss of environmental sensitivity that is reproducible across experiments. However, adaptive clones show reduced viability under starvation conditions, demonstrating an evolutionary tradeoff. These mutations are beneficial in an environment with a constant and predictable nutrient supply, likely because they result in constitutive growth, but reduce fitness in an environment where nutrient supply is not constant. Our results are a clear example of the myopic nature of evolution: a loss of environmental sensitivity in a constant environment is adaptive in the short term, but maladaptive should the environment change.

## Author Summary

When a population of organisms is faced with a selective pressure, such as a limiting nutrient, mutations that arise randomly may confer a fitness benefit on the individual carrying that mutation. If that individual reproduces before it is lost from the population, the frequency of that mutation may increase. Over time, many beneficial mutations will arise in a large population, but there are few high resolution experiments tracking the frequency of such mutations in an evolving population. We evolved populations of the baker's yeast in a constant environment in the presence of limiting amounts of sugar, and then used DNA sequencing to identify mutations that reached at least a 1% frequency in the population, and tracked them over time. We identified 120 mutations over three experiments, and determined that the genes and pathways that had gained beneficial mutations were largely reproducible across experiments, and that many of the mutations led to the loss of signaling pathways that usually sense a changing environment, allowing the cell to respond appropriately. When these mutant cells were faced with uncertain environments, the mutations proved to be deleterious. Environmental sensing must carry a fitness cost in a constant environment, but is essential in a changing one.

## Introduction

Adaptive evolution optimizes the fitness of organisms for their environment through the accumulation of beneficial mutations by natural selection [1]. While we understand much about the mechanisms by which natural selection operates, less is known about the beneficial mutation rate [2], and the genetic basis of adaptation [3]. Of particular interest is the spectrum of mutations that are adaptive in a specific environment, defined here as “adaptive strategy”. Through the use of experimental evolution, in conjunction with technological innovations such as candidate gene sequencing [4]–[8], cDNA, [9], [10] and tiling microarrays [11], [12], and whole genome sequencing of individual clones [13]–[16] and populations [17], [18], the field has recently gained significant insight into the genetic basis of adaptation. However, while candidate gene sequencing is certainly incomplete (though still instructive) in the picture it provides, even the identification of all mutations in individual clones does not reveal a complete representation of adaptation. Sequencing a handful of selected clones from an experiment provides only a microcosm of the adaptive mutational spectrum, while sequencing many clones from an experiment begins to resample the most prevalent lineages. By contrast, sequencing terminal clones from many different experiments (e.g. [14]) provides deeper insight into the convergence or divergence of the adaptive process, but is unable to capture evolution in action, including the clonal interference that occurs in the typically large populations used in microbial experimental evolution systems. To capture the dynamics of the adaptive process, as well as the mutational spectrum that accompanies it, it is necessary to sequence very large numbers of clones, possibly from many time points during an experiment, or instead to sequence entire populations as they evolve.

In large asexual populations, selection acts positively to increase the frequency of the lineages containing beneficial mutations, while competition between coexisting adaptive lineages reduces the overall rate at which beneficial mutation increase in allele frequency, a process termed clonal interference [19], [20]. Clonal interference occurs when beneficial mutations are sufficiently common to allow multiple adaptive lineages to expand in the population concurrently [21]. By deeply sequencing populations at multiple time points it is possible to not only identify mutations, but to also track the evolutionary dynamics of adapting lineages. Three studies published thus far have performed whole genome sequencing of evolving populations [17], [18], [22], identifying SNPs at as low as 5% allele frequency in the sequenced populations; in the first two of these studies, *E. coli* were evolved by serial transfer, effectively in a continuously varying environment. The second of these two studies [18], sequenced deeply enough that the allele frequencies of identified mutations over time could be tracked. However, it is likely that at ever-lower allele frequencies, there will be more observable beneficial mutations, most probably with smaller fitness effects. In the third of the studies, 40 replicate yeast populations were propagated by serial transfer for 1,000 generations, and sequenced every 80 or so generations [22], allowing allele frequencies to be able to be determined down to 10% allele frequency. To better enumerate the adaptive strategy under a particular environment and to gain a better quantitative measure of the extent of clonal interference, deeper sequencing is needed however, which will likely identify additional mutations at lower allele frequencies with which to better characterize the adaptive mutational spectrum.

Different environments likely result in different adaptive strategies, and many natural environments are variable and unpredictable, with irregular fluctuations in environmental parameters. Consequently, signaling networks evolved to enable organisms to be able to sense and respond to uncertain environments [23]. Signaling networks are ubiquitous across the Tree of Life, yet the question remains, “are functional signaling networks an essential feature of a well-adapted biological system?” Intracellular symbionts have undergone extensive genome reductions, likely due to relaxed selection in a setting that has few environmental perturbations. A major functional theme in these genome reductions is the loss of genes involved in signaling and genetic regulation [24], [25]. However, this loss is likely neutral gene degradation due to genetic drift rather than adaptive evolutionary processes [26].

We sought to determine if the loss of environmental sensitivity is a viable or indeed preferred adaptive strategy. A constant environment provides an opportunity for such a system to evolve, since environmental sensing is superfluous, and perhaps even carries a fitness cost. We characterized the adaptive strategy, and the dynamics of adaptive lineages of the budding yeast *S. cerevisiae* evolving in a constant environment by ultra deep genome- and population-wide sequencing of three parallel evolution experiments.

## Results

**Figure 1:**
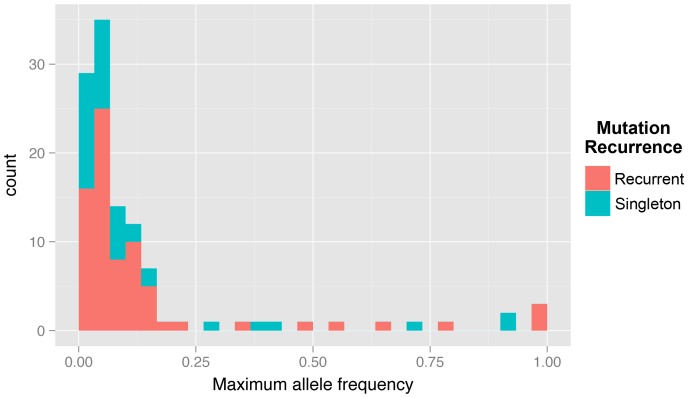
Histogram of maximum allele frequencies reached of all mutations discovered across the three experiments.

To determine the genetic basis of adaptation and the dynamics of arising mutations, we developed a novel population sequencing protocol, enabling the discovery of mutant alleles as well as their frequencies (Figure S1). We sequenced samples taken every ∼70 generations from three glucose-limited, chemostat-evolved populations of haploid S288c, named E1, E2 and E3, that have been described previously [12], [27]. Libraries were sequenced to 266–1046× coverage, and we employed an overlapping read strategy to reduce the sequencing error rate (Figure S1; Table S1). We also tagged each DNA fragment with a random barcode during library construction (Figure S1), enabling us to distinguish PCR duplicates from fragments that happened to map to the same genomic location; this reduced the apparent number of PCR duplicate reads by 100-fold. Our approach enabled the detection of mutations with an allele frequency as low as 1% (Figure 1), and in total, we discovered 117 mutations across all time points in the three experiments, of which 106 were in coding regions, affecting 51 genes, 19 of which were recurrently mutated. The mutations discovered, as well as their allele frequencies at each timepoint are given in Table S2.

### The dynamics of adaptation: Clonal interference and multiple mutations

**Figure 2:**
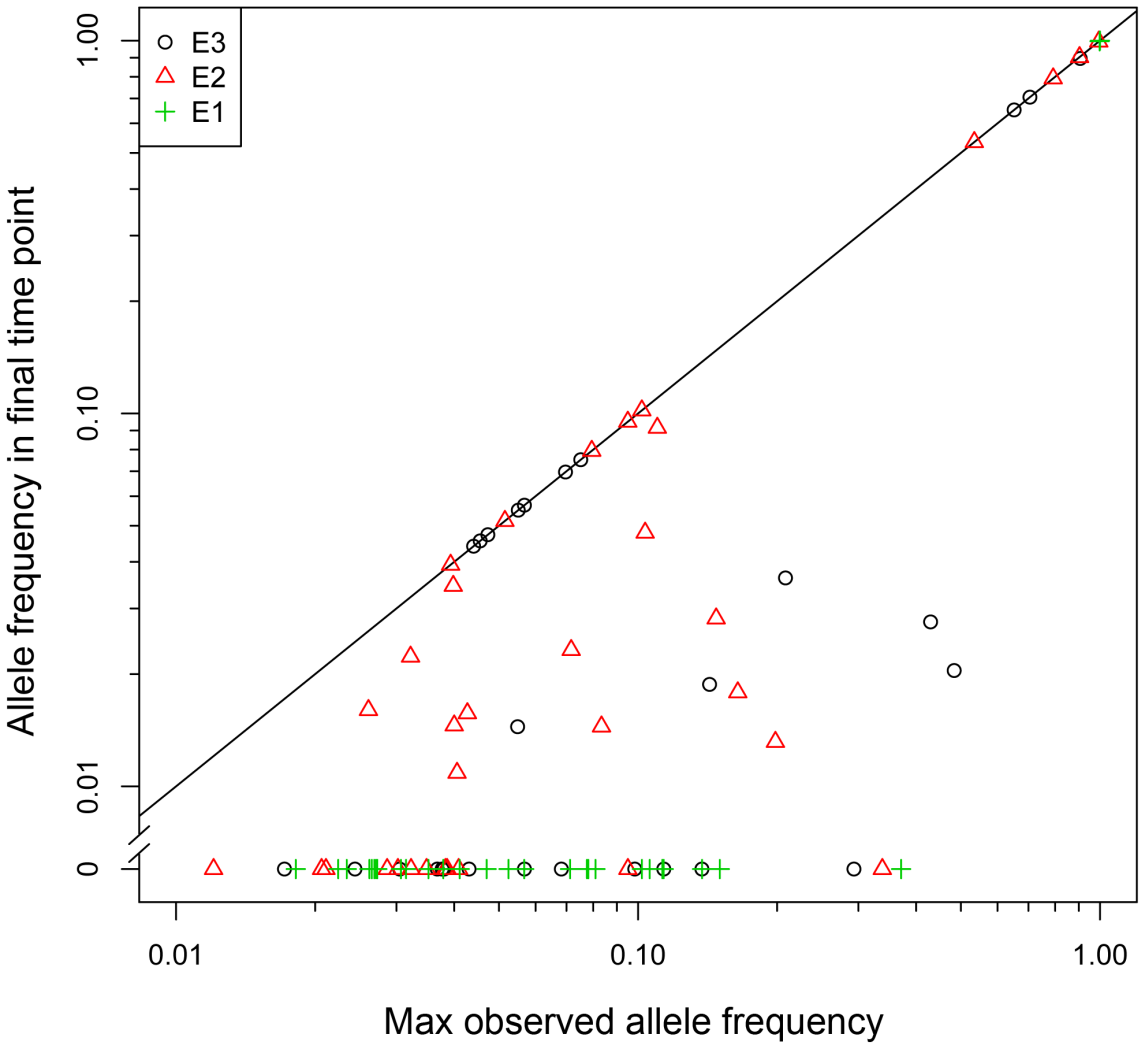
Clonal interference plays a prominent role in the dynamics of adaptation. Each point represents a mutation identified in one of the three experiments. Mutations that are at a lower allele frequency in the final time point than at an earlier time point (i.e. below the y = x line) have decreased due to interference from a competing lineage. Clonal interference affects 63% of all mutations, while 36% of mutations are driven to extinction due to clonal interference.

Our population sequencing shows that clonal interference plays a dominant role in all three experiments, as 74 of the identified mutations (63%) decrease in frequency following their maxima, and 42 of these mutations (57%) become extinct by the end of the experiment (Figure 2). These results agree with theoretical predictions [19], [28] and previous observations [29], and imply that even if a mutation rises to a level above our detection threshold, it is still likely to succumb to an expanding fitter lineage and eventually become extinct.

**Figure 3:**
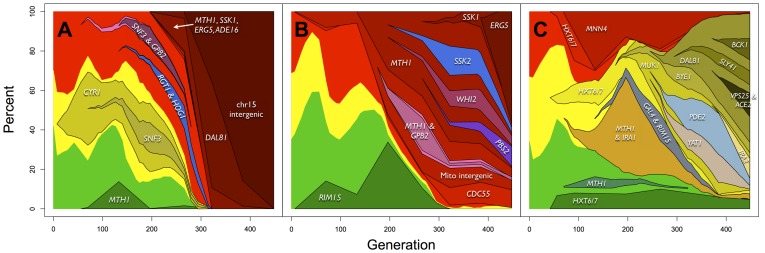
Dynamics and linkage of mutations above 10% allele frequency in (A) E1, (B) E2 and (C) E3. Mutations and frequencies were discovered from population sequencing, and linkage with other mutations and the fluorescent marker was determined by genotyping clones. *HXT6/7* frequency data in E3 are from [12]. The red, yellow and green indicate the frequencies of the fluorescent makers (compare to Figure 1 of [12]) with lineages within those differently marked subpopulations originating from within them.

Evolution under conditions that promote clonal interference is also predicted to promote the accumulation of multiple beneficial mutations within an adaptive lineage before the first mutation can sweep [21]. We genotyped clones to determine the linkage of mutations above 10% frequency, and find that 91% of these mutations coexist in a clone with one or more other mutations. This value is an underestimate, since most mutations (67%) never reach 10% frequency and thus were not analyzed for linkage. While from sequencing data alone we cannot unequivocally label a mutation as beneficial versus neutral, recurrent independent mutations (see below and Figure S2) are likely to be beneficial. By this definition, all lineages that we were able to define by genotyping carry at least 1 beneficial mutation (Figure 3). Furthermore, the “winning” lineages occupying the largest proportion of the final population carry at least three beneficial mutations, and at least five mutations total (Figure 3). An exceptional case is E1, where six mutations occur in close succession (four of which are genes that we observe as recurrently mutated) and result in what appears to be a complete selective sweep (Figure 3a). These data indicate that multiple beneficial mutations – often occurring in close succession on what appears to have been a wild-type background – are necessary for a lineage to be successful. However, having multiple mutations is not sufficient for a lineage's success; for example, three lineages in E1, each with two mutations, become extinct due another lineage sweeping (Figure 3a). Furthermore, almost two thirds (49/76) of recurrent, and thus likely beneficial mutations never reach 10% frequency. The dynamics of adaptation suggest the “survival of the luckiest”, where for a new beneficial mutation to reach a high frequency, it must occur on a background that already has multiple other beneficial mutations [29]. This makes predicting the outcome of adaptive evolution difficult since the fixation probability of a beneficial mutation is no longer deterministic and proportional to the selection coefficient, but is also dependent on the genetic background on which the mutation occurs, which is distinctly a chance event. Our data show unequivocally that clonal interference between lineages carrying multiple beneficial mutations defines the dynamics of adaptation.

### The genetic basis of adaption: Loss of signaling pathways

**Figure 4:**
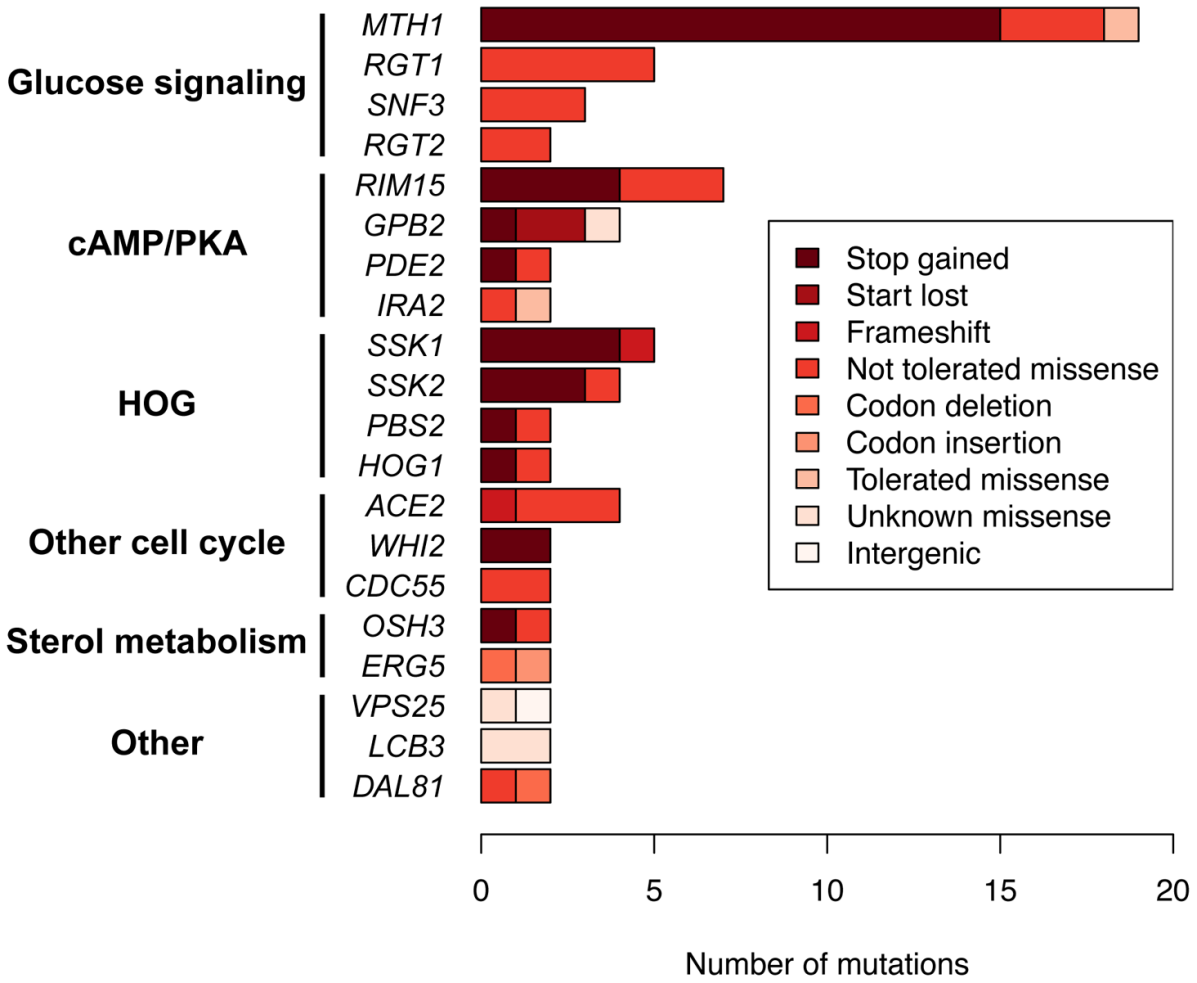
Identity, severity and function of recurrently mutated genes across all experiments, grouped by pathway. Only genes with two or more identified mutations are included; bars are colored according to the predicted severity of each mutation on protein function.

**Figure 5:**
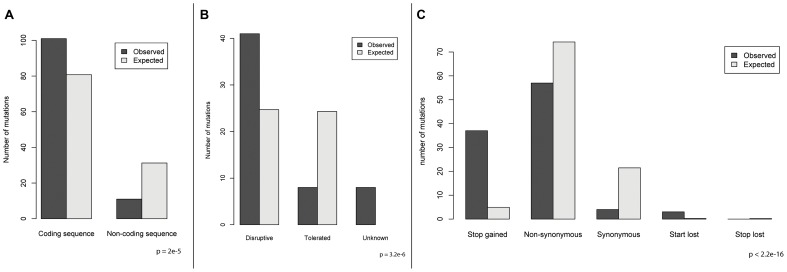
Enrichment of mutation categories relative to the expectation. Expectation was calculated empirically assuming random mutation across the genome, and significance of enrichment was determined using a chi-squared test. A) Coding versus non-coding mutations. B) Mutations that are predicted to be disruptive of protein function versus mutations predicted to not affect protein function. C) Enrichments within coding mutations.

We sought to understand the adaptive strategy of yeast growing in a constant environment by categorizing the genes in which mutations had occurred. Grouping recurrently mutated genes by pathway, we find that 53% of these mutations across all experiments reside in genes which function in three major signaling pathways: glucose signaling and transport, cyclic adenosine monophosphate/protein kinase A (cAMP/PKA) and the high osmolarity glycerol (HOG) response pathway (Figure 4), and these pathways have statistically enriched GO terms (Table S3). To further characterize the adaptive strategy, we characterized mutations by their predicted consequences. We found that the majority (73%) of mutations are predicted to disrupt protein function, with nonsense mutations being enriched by 7.6-fold (p<2.2e-16) (Figure 5). Together, these data suggest that the general adaptive strategy in a constant environment is the loss of signal transduction pathway function (Figure 4). For the glucose signaling pathway, disruptive mutations in *MTH1* and *RGT1* lead to constitutive expression of the glucose transporter (HXT) genes [30], [31], which increases the amount of glucose that is able to enter the cell, facilitating growth and providing a selective advantage [27]. The cAMP/PKA pathway positively responds to glucose in wild-type cells leading to growth [32]; disruptive mutations in the three recurrently mutated repressors *GPB2*, *IRA2* and *PDE2* would cause constitutive pathway activation and growth, while loss of function in *RIM15* (the second most mutated gene, with 7 mutations observed), which is repressed by the PKA pathway, is akin to having increased PKA activity through that downstream path. Rim15 function is involved in the establishment of stationary phase [33] – presumably loss of the ability to enter stationary phase must be beneficial in the constant chemostat environment. The HOG pathway mediates transcriptional response to hyper-osmotic stress and also causes a brief growth arrest [34], so the observed disruptive mutations in pathway activators would be expected to eliminate this response. All five HOG pathway mutations we genotyped occur in lineages with pre-existing *MTH1* or *RGT1* mutations, (Figure 3a–b), suggesting potential positive epistasis between the HOG and glucose signaling pathways.

To assess the extent of parallel adaptation we examined the overlap in genes and GO terms between experiments. E1, E2 and E3 share 50%, 61% and 21% of their mutated genes with one of the other experiments, with E1 and E2 having the most sharing. *MTH1*, *RIM15* and *GPB2* are mutated in all three experiments, with *MTH1* being the most frequently observed mutated gene, having 19 independent mutations observed. We grouped enriched GO terms that share edges into GO networks to eliminate redundant GO terms and determined that E1 and E2 share all GO networks with each other (Table S4). E3 has overlap with the other two experiments, with 3 of 6 networks shared with both E1 and E2. The GO network overlap suggests that the replicate experiments followed similar functional trajectories, with the underlying mutations broadly impacting similar biological processes in all experiments, namely the disruption of environmental sensing and signal transduction.

### An evolutionary trade-off by antagonistic pleiotropy

**Figure 6:**
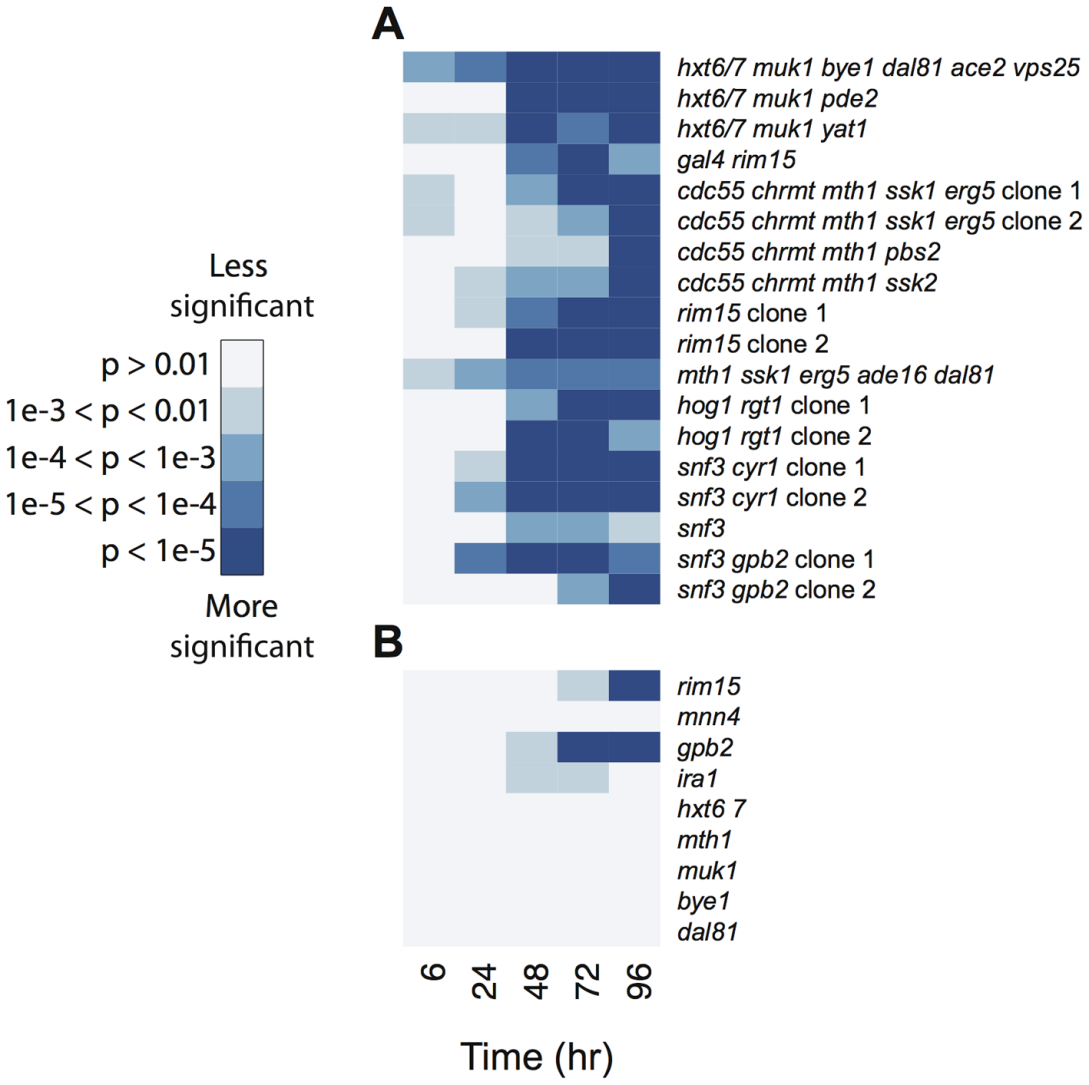
Reduction in cell viability as a function of time in (A) clones isolated from the chemostat experiments and (B) strains carrying a single mutation from E3. The deeper the blue color, the more significant the reduction in cell viability compared to a wild-type strain. Multiple independent clones with the same known genotype are indicated.

We have shown that loss of environmental sensing through disruptive mutations in signaling pathways is adaptive in a constant environment. As signaling pathways make organisms robust to environmental changes, we hypothesized this loss would have a fitness cost in environments where nutrient availability was not constant. We thus subjected 18 clones containing mutations in one or more signaling pathway to starvation conditions. All 18 clones lost viability more rapidly than wild-type (Figure 6a). To understand which mutations were causing decreased viability, we assayed nine strains containing single mutations from E3 [27] for premature cell death, and found that mutations in or downstream the cAMP/PKA pathway (4 of 9 mutations assayed) showed significantly lower cell viability during starvation (Figure 6b). Of these, we have previously shown that mutations in 3 of these 4 genes are beneficial alone in a glucose limited chemostat [27]. Thus, our results suggest that the adaptive strategy utilized by yeast in the constant chemostat environment is maladaptive in an environment where nutrients are not constant, indicating that there is an evolutionary trade-off due to antagonistic pleiotropy (e.g. see [35]).

## Discussion

### The dynamics of adaptive evolution

We have previously used fluorescent markers to track subpopulations during adaptive evolution in a constant environment [12], and observed clonal interference in each of the 8 experimental populations that we evolved, in concordance with theoretical expectations. In this work, we have greatly expanded upon this, by performing ultra deep whole genome, whole population sequencing at each of 8 timepoints across 3 of these experimental evolutions. In addition to allowing us to identify mutations at an allele frequency as low as 1%, these population sequence data also provide us with direct estimates of the frequency trajectories of the mutations. Of the 3 experiments, only one resulted in a fixation event (E1, where 4 mutations in the same lineage were fixed by the final time point at 448 generations). By contrast, most mutations that enter the population were at a lower frequency than their maximum by the end of the experiment, and indeed more than a third had gone extinct. In most cases, mutations that were subject to clonal interference were in genes that were recurrently mutated (53 out of 74 (72%)), and of those mutations that went extinct, the majority were also in genes that were recurrently mutated (32 out of 42 (76%)). Thus, clonal interference clearly plays a major role in these populations in shaping their eventual composition, with many beneficial mutations in the population going extinct during the evolution. A recent study which also used sequencing of populations undergoing experimental evolution [18] did not observe such a great extent of clonal interference, though in their experiments they only could detect mutations that reached a 5% allele frequency. In our data, of the 74 mutations we detected that were at a lower frequency by the end of the experiment than their maximum frequency (i.e. were subject to clonal interference), 38 (51%) had a maximum frequency of less than 5%. Thus, deeper sequencing is able to provide significantly more insight into the process of clonal interference. We observed 35 mutations in genes that were recurrently mutated that failed to reach a 5% frequency in the experiment, though we only identified 2 additional recurrently mutated genes by being able to get to allele frequencies lower than 5% (*OSH3* and *LCB3*). There were 19 mutations that did not reach an allele frequency of 5% that were in genes that were not recurrently mutated – further experimentation to determine whether these mutations are adaptive, and/or even deeper sequencing would be required to confidently extend the adaptive mutational spectrum. We also observed that multiple mutations prevail, with all of the lineages that we detect as present in our populations at the end of the experiment carrying more than one mutation, with at least two predicted to be beneficial.

It is an open question as to how many lineages with beneficial mutations actually existed within the population – there are few empirical estimates of the beneficial mutation rate, and those that do exist are based on a relatively modest number of observed mutations. One estimate, based on mutations that fixed in *Pseudomonas fluoresecens*, is 3.8e-8 per cell division [36]. If that were similar to the beneficial mutation rate in yeast, then with a population of 1e9 growing for 448 generations, we might expect as many as 17,000 beneficial mutations to occur within any one of our experiments. Most of these would not be expected to establish – if we assumed that ∼10% establish (roughly similar to an average 10% fitness effect), then 1,700 lineages with beneficial mutations would have established in a given experiment. By contrast, Shaw et al [37], analyzing mutation accumulation lines in *A. thaliana*, found that approximately half of all mutations observed were beneficial. In yeast, also using mutation accumulation lines, Hall and colleagues have estimated that between 5% and 13% of mutations are beneficial [38]–[40]. With a per base pair mutation rate on the order of ∼1e-10 [41] and a genome size of 12e6, the number of cells estimated per generation to have a mutation is around 1 in 1,000. If 10% of mutations are beneficial, then 1 in 10,000 per generation may receive a beneficial mutation. Thus, in our experiments, we might expect as many as 50 million beneficial mutations to occur over the 448 generation time course, with ∼5 million establishing. While these are estimates based on relatively small number of mutations in mutation accumulation lines, even if they are over estimated by 2 orders of magnitude, it is clear that sequencing of even hundreds of randomly selected individual clones (which will likely represent a few, prevalent lineages), or even deep population sequencing will not be able to fully characterize the spectrum of beneficial mutations, nor determine an accurate estimate of their fitness effects. While to our knowledge this study is the deepest sequencing yet performed on experimentally evolving populations, it may only represent the tip of the potential adaptive iceberg (though this is likely the most important part, as these mutations likely drive the evolutionary process), while our previous work [12] was only the tip of the tip. New, higher throughput approaches, and rational ways of identifying and selecting independent lineages are clearly needed to fully understand this most fundamental of biological processes.

### Adaptive strategy and parallel evolution in a constant environment

**Figure 7:**
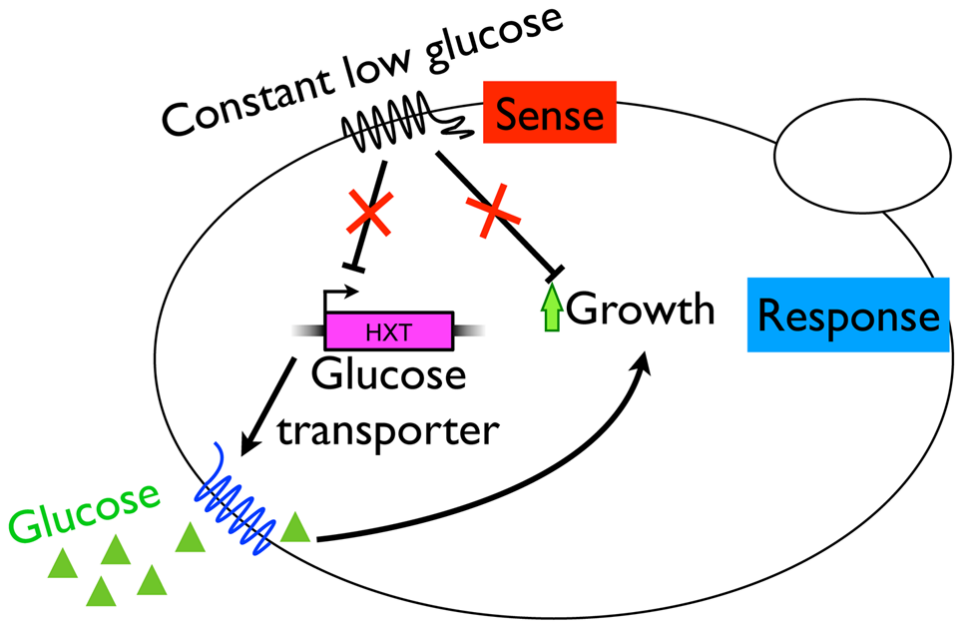
A model for adaptive strategy in the constant, glucose-limited environment of the chemostat. The accumulation of beneficial mutations disruptive of signaling networks leads to the decoupling of sensing from response and the loss of environmental sensitivity. Loss of control of cAMP/PKA pathway function eliminates some of the normal checks required to pass START A, likely to a shortened G1 and constitutive cell division. Likewise, loss of repressors of glucose transporter transcription leads to their constitutive activation, likely enabling the cell to sequester more glucose, leading to increased growth and division.

We observed the parallel evolution of mutations that disrupt one or more of three major signaling pathways responsible for sensing environmental stimuli and responding by governing growth rate. We propose a model for the adaptive strategy in constant, nutrient-limited environments (or at least in a glucose limited environment) (Figure 7), wherein constitutive commitment to cell division is beneficial, and thus mutations that result in unrestrained cell division are adaptive as long as the growth rate does not exceed the influx rate of nutrients into the system. By and large, these mutations are loss of function mutations. We consider the mutations in these pathways to be decoupling the sense and response to environmental stimuli, leading to an adaptive loss of environmental sensing in a constant environment. In contrast, these mutations are maladaptive in environments where nutrient abundance is not constant, such as when going through a boom and bust cycle from high glucose into starvation conditions. This may be due to depletion of the cell's reserve nutrient supply or the inability to enter a quiescent state, leading to cellular death. This adaptive loss of environmental sensitivity is a powerful example of how evolution is myopic: by evolving strategies to cope with a constant and predictable environment, genes and pathways are disrupted that would be necessary for survival when cells are confronted with an uncertain environment. It is noteworthy that the clones characterized in Wenger et al [35], also evolved in an aerobic glucose limited environment, were also more fit under a diverse set of other carbon limited environments, suggesting that their adaptive strategy also translated to other constant environments. Whether this strategy is widely applicable under an array of constant environments with different nutrient limitations remains to be determined through additional experimentation. However, recent analysis of experiments in bacteria have verified the idea that loss of function mutations can be a general strategy for adaptive evolution [42].

### Broader impacts and antagonistic pleiotropy

From a broad viewpoint, the adaptive strategy of loss of environmental sensitivity that we observed is similar to the strategy tumor cells use to proliferate. Cancer is an evolutionary process of clonal selection [43]–[45], and it is beneficial for the cells to replicate as fast as possible through the accumulation of mutations in oncogenes and tumor suppressor genes, many of which are in the homologous Ras/cAMP/PKA pathway that is recurrently mutated in our experiments [46], [47]. While the external environment humans face is dynamic and unpredictable, the human body has evolved to maintain homeostasis, exemplified by the near constant concentration of blood glucose [48]. Such mutations also come with trade-offs – when faced with an uncertain environment, many of the mutations show antagonistic pleiotropy (AP). In our data, three single mutants that we tested had a reduced fitness in the starvation environment, for which we have previously demonstrated fitness gains in the chemostat environment where they were selected – clearly cases of AP. For the multiple mutants that we tested, the loss of fitness could be due to AP, or alternatively result from a prior hitchhiking event in the evolved environment of a mutation that is deleterious in the starved environment. This mutation accumulation hypothesis (MA) is considered as an alternative to antagonistic pleiotropy when an evolved lineage shows fitness trade-offs. The fact the many, if not all of the mutations in our multiple mutants are in genes or pathways that are recurrently mutated in our chemostat evolutions makes MA seem a less likely explanation than AP. Indeed, previous experiments using *E. coli* evolving by serial transfer [49] showed that the rate of loss of unused functions in parallel evolving populations was consistent with AP, rather than MA, suggesting that AP may be widespread, and that when evolving in a consistent (though not necessarily constant) environment that due to the fitness cost of unneeded pathways, that there is a use it or lose if effect [50]. It has been shown using the yeast deletion collection that AP is indeed widespread, with approximately 20% of the collection of non-essential gene deletions being more fit under one of the tested conditions [51]. It is also of note that in evolving *E. coli* strains, mutations that result in a loss function of the sigma factor encoded by *rpoS* (which is involved in the general stress response) are frequently selected [52]. These mutations frequently exhibit AP, being detrimental under conditions where there is stress, the response to which needs to be balanced with growth (see [53] for review). Most of the AP examples thus provided are loss of function mutations (either from systematic gene deletion projects, or from sequencing beneficial mutations arising during experimental evolution), but a systematic catalog of AP effects of large numbers of beneficial mutations has not yet been generated. It will also be interesting to determine how clones with beneficial mutations that exhibit AP can perform adaptive escape when allowed to evolve afresh in an environment in which their previously beneficial mutations are now deleterious.

## Materials and Methods

### Source of evolved samples

All population samples in this study have been previously described [12]. Briefly, three strains of haploid S288c that are isogenic, except that each constitutively expresses a different fluorescent protein (GFP, YFP or DsRed), were seeded in equal quantities in a 20 mL chemostat device. Each population was evolved for 448 generations at steady state under glucose limitation (0.08%) at a dilution rate of 0.2 h^−1^. During this evolution, the proportions of the three colored lineages were tracked using flow cytometry, and population samples were archived under deep freeze in glycerol at −80°C at regular intervals. Wild-type ancestral strain GSY1136 was also used as a reference for sequencing.

### Sequencing library construction

Illumina sequencing libraries were made directly from glycerol stocks of the original population samples, as well as the wild-type ancestral strain (GSY1136). Stocks were melted, and genomic DNA was extracted from 500 µl of each stock using Zymo Yeast Genomic DNA columns. The Nextera library prep kit (Epicentre) was used to construct the libraries, starting with 25–50 ng of genomic DNA. The tagmentation reaction was performed in LMW Reaction Buffer at 55°C for 10 minutes. The resulting tagged DNA was subjected to PCR using the Nextera PCR enzyme (Epicentre) under the following conditions: 72°C for 3 min, 95°C for 30 sec; 9 cycles of 95°C for 10 sec, 62°C for 30 sec, 72°C for 10 sec; final extension at 72°C for 1 min. A shortened extension time was used to bias the amplification of short fragments in order to maximize the proportion of bases being sequenced twice with overlapping paired-end Illumina reads. A modified Adapter 2 with a random hexamer barcode of sequence 5′-CAAGCAGAAGACGGCATACGAGATNNNNNNCGGTCTGCCTTGCCAGCCCGCTCAG-3′ (PAGE-purified, IDT Technologies) was used during the PCR for the population samples, while the standard Nextera Adapter 2 was used for GSY1136. No size selection was performed on the libraries, although they were concentrated through a Qiagen MinElute column. The same GSY1136 library was spiked into all 24 population libraries at a molar rate of 5%. The resulting libraries were sequenced on one lane apiece of 2×101 bp plus a 6 bp index read on the Illumina Hi-Seq 2000. In addition, two independent libraries from the same genomic DNA of GSY1136 were sequenced on one Hi-Seq lane apiece.

### Sequencing data mapping and pre-processing

An overview of the sequencing analysis pipeline used to identify variants is given in Figure S3. The wild-type GSY1136 library that was spiked into each population sample was extracted with the exact tag ATCTCG using a modified version of the Fastx Barcode Splitter (http://hannonlab.cshl.edu/fastx_toolkit/index.html). Nextera adapters were trimmed off the 3′ read ends with Cutadapt v0.9.4 [54] supplied with the Nextera adapter sequence and default parameters except -m 15. The resulting FASTQ files were culled of any reads that occurred in only one read of the pair. Paired-end reads were mapped to a custom S288c reference genome with BWA (bwa-short) v0.5.9-r16 [55] using default parameters plus -I -q 10, and a sorted BAM file was created with Picard v1.45 FixMateInformation (http://picard.sourceforge.net).

The custom genome was constructed as follows: single end Illumina reads of a different ancestral wild-type strain (GSY1135) from a previous study [27] were mapped to the S288c reference sequences from the *Saccharomyces* Genome Database (SGD; http://www.yeastgenome.org/; downloaded 2/24/2011). SNPs were called with the GATK v1.0.5777 UnifiedGenotyper [56], [57], and a FASTA reference sequence was constructed that incorporated these SNP calls using the GATK FastaAlternateReferenceMaker.

The population data were culled of PCR duplicates using a modified version of Picard MarkDuplicates. In this program, the random hexamer barcodes were used in addition to the mapping coordinates to decide if a pair of reads was a PCR duplicate. Specifically, if more than one read pair had the same mapping coordinates in addition to the same hexamer barcode, only the pair with the highest mapping quality was retained for further analysis.

The in-lane spike-in of wild-type library was used to recalibrate the base qualities of the population data from the same lane. To achieve this, GATK CountCovariates and TableRecalibration were called on each lane of the wild-type data separately, using a variant mask for the CountCovariates step created by Samtools v0.1.16 [58] mpileup. Recalibration was visualized as successful as visualized by AnalyzeCovariates. The covariate file from the wild-type recalibration was then used as input for TableRecalibration on the population data from the same lane. Proper recalibration was assessed once again by AnalyzeCovariates.

A custom Java program was written to identify the bases in each library fragment that were sequenced twice by overlapping read pairs. This analysis was applied to both population and wild-type data, and the overlap information was stored in the custom “ZO” tag of the BAM file. A Python script implementing PySAM v0.5 (http://code.google.com/p/pysam/) was used to calculate the allele counts for each position in the reference genome, and the following filters were applied: uniquely mapping reads only, base quality score greater than 19 required, and only bases sequenced twice that were concordant in base identity between the two reads were retained.

### SNP calling and filtering

Population SNP calls were made by comparing the allele counts in each population sample for each genomic position to the counts of the same allele and position from the wild-type data. This comparative approach filtered out any position that had false positive SNP calls due to positional effects, such as mapping or systematic sequencing errors. First, a merged wild-type file was created by combining all the spike-in control wild-type data with the two independently sequenced wild-type files. Second, only non-reference alleles that had both an allele count of at least 2 and a larger frequency in the population sample than the wild-type were retained. Third, a one-tailed Fisher's Exact Test was used to calculate if the number of non-reference alleles out of all alleles at a site was significantly greater in the population data than in the wild-type data for the same allele. These p-values were FDR corrected using the Benjamini and Hochberg method [59], and only sites with a q-value less than 0.01 were retained.

The following heuristic post-hoc filters were applied to the set of SNPs: 1) SNPs with a maximum frequency that was greater than the largest color proportion, plus 0.1, for the appropriate time point were removed (color frequency data from [12]). This removes any SNP that rose to a higher frequency than the highest color, which is not possible, unless identical SNPs arose in different colored populations. 2) Any SNP that was significant in the first time point was removed. This is because even if a new mutation present at the start of the experiment conferred a relative fitness of 2, that mutation would not be detectable in our assay in the first sampled generation. 3) Any site that was not deemed callable was removed. Callability was determined empirically with the GATK CallableLociWalker (-frlmq 0.01 -minMappingQuality 2) on the relevant population data, as well as the merged wild-type data. 4) Sites that had greater than 5% non-reference alleles in the merged wild-type data were removed. These sites were largely systematic errors. 5) SNPs where the read position of the variant allele did not vary were removed. This was defined as a read position standard deviation lower than one. 6) SNPs that had a mapping quality bias between reads containing the reference and variant alleles were removed, as calculated by a Bonferroni-corrected Mann-Whitney U test on mapping qualities.

### Variant confirmation, lineage determination and inference of effects

Mutation allele frequencies were validated against a set of known mutation frequencies for experiment C1 (Figure S4) with data from [12], [27], as well as the fluorescent protein reporter frequencies for all experiments (Figure S5). All putative SNPs with a maximum allele frequency greater than 10% were confirmed by Sanger sequencing, except for the chr16:581589 mutation in experiment E2, which we were unable to amplify by PCR. While no effort was made to comprehensively catalog indels, Sanger sequencing of putative SNPs revealed six indels, which in every case were due to mapping errors of true indels near the ends of reads.

Co-occurrence of SNPs was determined by Sanger sequencing clones picked from the relevant time points for mutations greater than 10% allele frequency. The effect of each SNP (non-coding, synonymous coding, non-synonymous coding, etc.) was established with SNPeff v2.0.3 (http://snpeff.sourceforge.net/). The permissiveness of all missense mutations was calculated using SIFT [60] with default parameters.

To create the lineage dynamics plots, allele frequency data were plotted assuming linear expansion or contraction between primary data points. Since the allele frequency data were of lower resolution than the flow cytometry data (8 vs 47 time points), sometimes the inferred linear extrapolation between frequency data points resulted in an allele frequency greater than the color frequency. In these cases, the extrapolated allele frequencies were reduced to fit within the bounds of the color frequencies. Note, this fitting was performed for extrapolated points only; primary allele frequency data remained untouched.

### Mutation effect enrichment analysis

All mutations discovered across the three experiments were divided into the following coding mutation effect categories: stop gained, start lost, stop lost, non-synonymous and synonymous. The sum of mutations within these categories was compared to the expectation using a chi-squared test. The expectation was calculated empirically by assuming random mutation throughout the genome; i.e. all possible mutations in the genome were made *in silico*, and the effect of the mutation was assigned to one of the categories above. The expected proportion of each category was calculated as the total for each category out of all possible mutations, and this proportion was multiplied by the total number of mutations discovered to get the expected number of mutations for each category. The same analysis was performed for coding versus non-coding mutations.

To find an enrichment of disruptive versus tolerated mutations, the totals of the stop gained, start lost, stop lost and disruptive non-synonymous categories were summed into the “disruptive” meta-category, and the synonymous, tolerated non-synonymous and non-coding mutations were summed into the “tolerated” meta-category. The SIFT predictions were used to classify non-synonymous mutations as either disruptive or tolerated. Expectations for disruptive or tolerated non-synonymous mutations were calculated empirically by summing the SIFT effect of all possible mutations for a particular protein.

### Cell death experiment

Cell viability was quantified under starvation conditions using propidium iodide (PI) and flow cytometry similar to [61], in biological triplicate. Overnight cultures in 1.2 mL YPD were grown unshaken in deep-well 96 well plates at 30°C. Cultures were spun down, aspirated, and resuspended in 1.2 mL sterile water, and then diluted 1∶3 into a minimal medium described previously [12] supplemented with 2% glucose. The cultures were left undisturbed at 30°C between time points. Cell viability was measured at regular intervals post-inoculation by mixing the cultures and diluting 50 µL of culture into 250 µL water containing 250 µg PI, following by analysis by flow cytometry. The proportion of viable cells was calculated as PI-negative cells out of total cells analyzed. Significantly different viability was calculated with a two-tailed t-test between each mutant strain and wild-type at each time point. Cell viability based on PI staining was validated by colony forming unit analysis on a subset of the strains analyzed.

### Gene Ontology enrichment

Gene Ontology (GO) biological process enrichments of coding mutations for each experiment were calculated using GO::TermFinder [62] at SGD with default options except “Feature Type” set to “ORF” and dubious ORFs disqualified from the analysis. For the reproducibility analysis, GO terms sharing edges were grouped into networks and GO networks were considered shared between experiments if they had at least one shared GO term.

### Accession numbers

All Illumina sequencing data are available from the NCBI Sequence Read Archive with accession number SRA054922.

## Supporting Information

Figure S1 Diagram of sequencing library preparation and sequencing strategy. Two improvements were made to the Nextera library preparation to facilitate the detection of low frequency SNPs. First, random hexamer barcodes were added to Adapter 2 to reduce the observed rate PCR duplicates. PCR duplicates are a problem for Nextera libraries sequenced to high coverage because the transposase used for library construction has an insertion bias, which leads to independent genomic DNA fragments mapping to the same genomic location. Second, the insert size of the library was biased towards short fragments by reducing the PCR extension time. This caused most bases per genomic DNA fragment to be sequenced twice with overlapping read pairs, which reduced the error rate of sequencing.
(PDF)

Figure S2 Allele frequency trajectories of all mutations discovered in A) E1, B) E2 and C) E3. Thick dashed lines show the proportions of each fluorescent protein reporter. Solid thin lines are mutations in genes that are recurrently hit with mutations, and thus command more confidence as driver mutations. Dashed thin lines are mutations in genes hit once.
(PDF)

Figure S3 Diagram of the analysis pipeline used to call SNPs from population sequencing by converting raw data to allele counts and allele counts to SNP calls. Actions performed on data are in boxes, with programs used in parenthesis, if applicable.
(PDF)

Figure S4 Validation of mutation allele frequency estimates using known mutations from E3. Solid lines are allele frequencies from this study. Dashed lines are frequencies of the same mutation as determined by either allele-specific quantitative PCR or quantitative sequencing. The time points that have data are different between the population sequencing and the validation, which contributes to the differences observed.
(PDF)

Figure S5 Validation of mutation allele frequency estimates using known mutations carried by fluorescent reporter strains in A) E1, B) E2 and C) E3. Each strain carries a single SNP, except the DsRed strain used in E1. Solid lines indicate the proportions of each fluorescent protein reporter as determined by flow cytometry. Dotted lines show the allele frequency of the SNP carried by each fluorescent strain.
(PDF)

Table S1 Sequencing coverage of each sequencing library. Numbers are average fold sequencing coverage. The coverage from bases sequenced twice by overlapping read pairs are shown (OL), as well as bases sequence only once (non-OL). Only OL bases were used for the analysis to call SNPs.
(PDF)

Table S2 List of mutations discovered in each experiment. Once a mutation was discovered by the analysis pipeline, the frequency of the mutation was pulled from other time points to complete the allele frequency trajectory.
(PDF)

Table S3 Gene Ontology (GO) biological process enrichments for each experiment. All genes hit with at least one mutation were the input for the GO enrichment analysis, and only GO terms with an FDR-corrected p-value of 0.01 or less are shown.
(PDF)

Table S4 GO terms were organized into networks and overlap between GO networks was calculated to assess the functional reproducibility of adaptation between experiments (see methods). GO terms in bold/italic/underline are terms that define a network, and that are shared between all three experiments. GO terms in italic/underline define the network and are shared only between E1 and E2.
(PDF)
