# Supplementary figures and images for: Whole Genome, Whole Population Sequencing Reveals That Loss of Signaling Networks Is the Major Adaptive Strategy in a Constant Environment
Source: PLoS Genet. 2013 Nov 21;9(11):e1003972. doi: 10.1371/journal.pgen.1003972 (PMC3836717; doi:10.1371/journal.pgen.1003972)

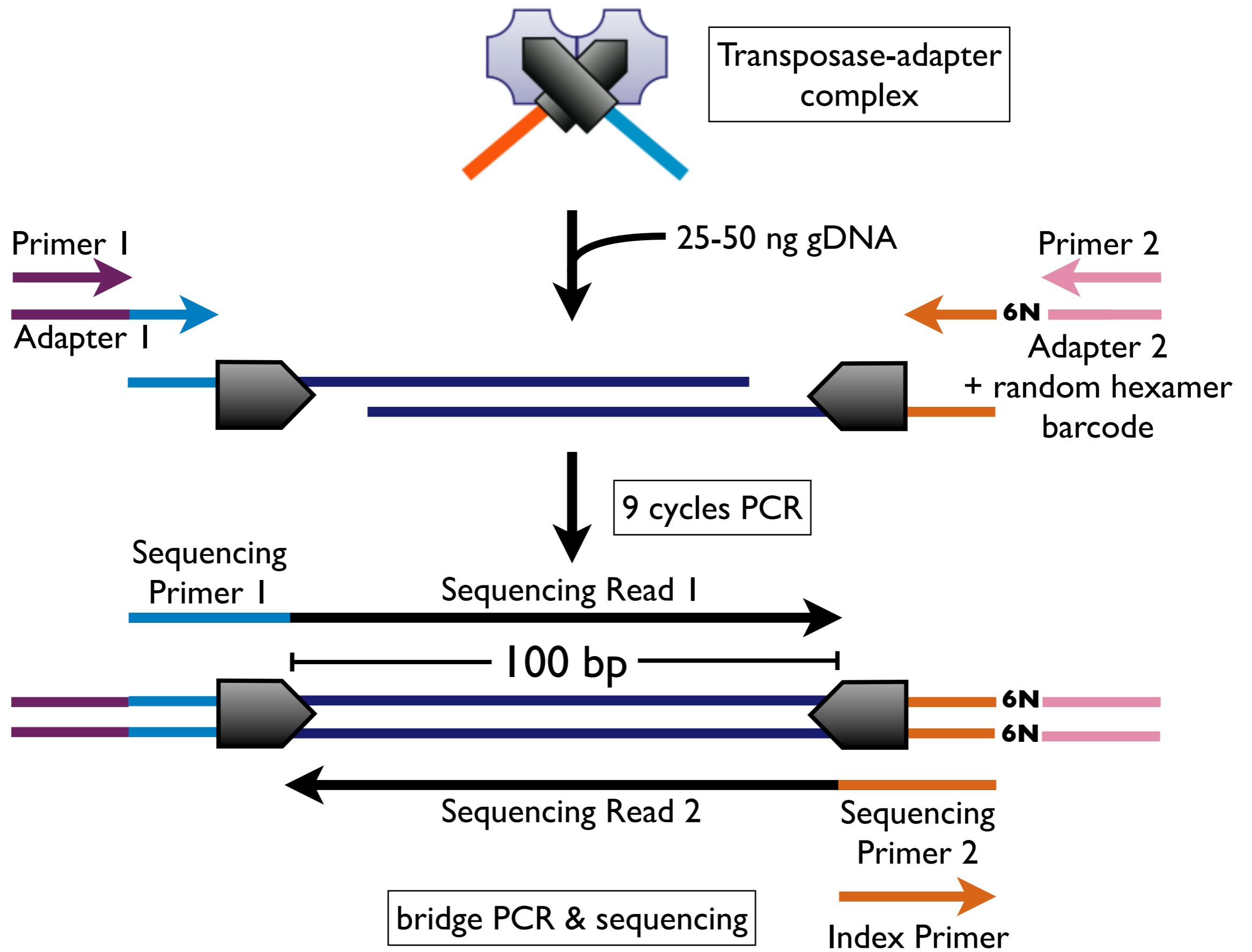

Supplementary Figure 1.

Supplement: Figure S1 — Diagram of sequencing library preparation and sequencing strategy. Two improvements were made to the Nextera library preparation to facilitate the detection of low frequency SNPs. First, random hexamer barcodes were added to Adapter 2 to reduce the observed rate PCR duplicates. PCR duplicates are a problem for Nextera libraries sequenced to high coverage because the transposase used for library construction has an insertion bias, which leads to independent genomic DNA fragments mapping to the same genomic location. Second, the insert size of the library was biased towards short fragments by reducing the PCR extension time. This caused most bases per genomic DNA fragment to be sequenced twice with overlapping read pairs, which reduced the error rate of sequencing. (PDF) [file pgen.1003972.s001.pdf]

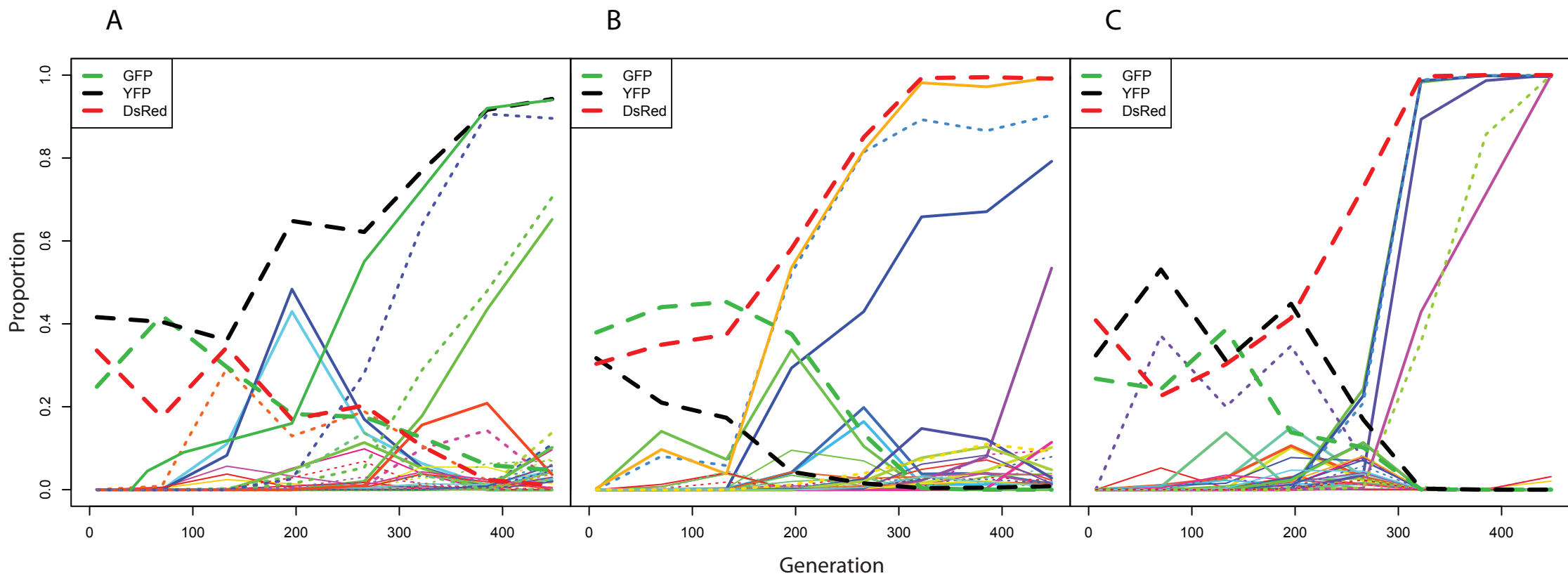

**Supplementary Figure 2**

Supplement: Figure S2 — Allele frequency trajectories of all mutations discovered in A) E1, B) E2 and C) E3. Thick dashed lines show the proportions of each fluorescent protein reporter. Solid thin lines are mutations in genes that are recurrently hit with mutations, and thus command more confidence as driver mutations. Dashed thin lines are mutations in genes hit once. (PDF) [file pgen.1003972.s002.pdf]

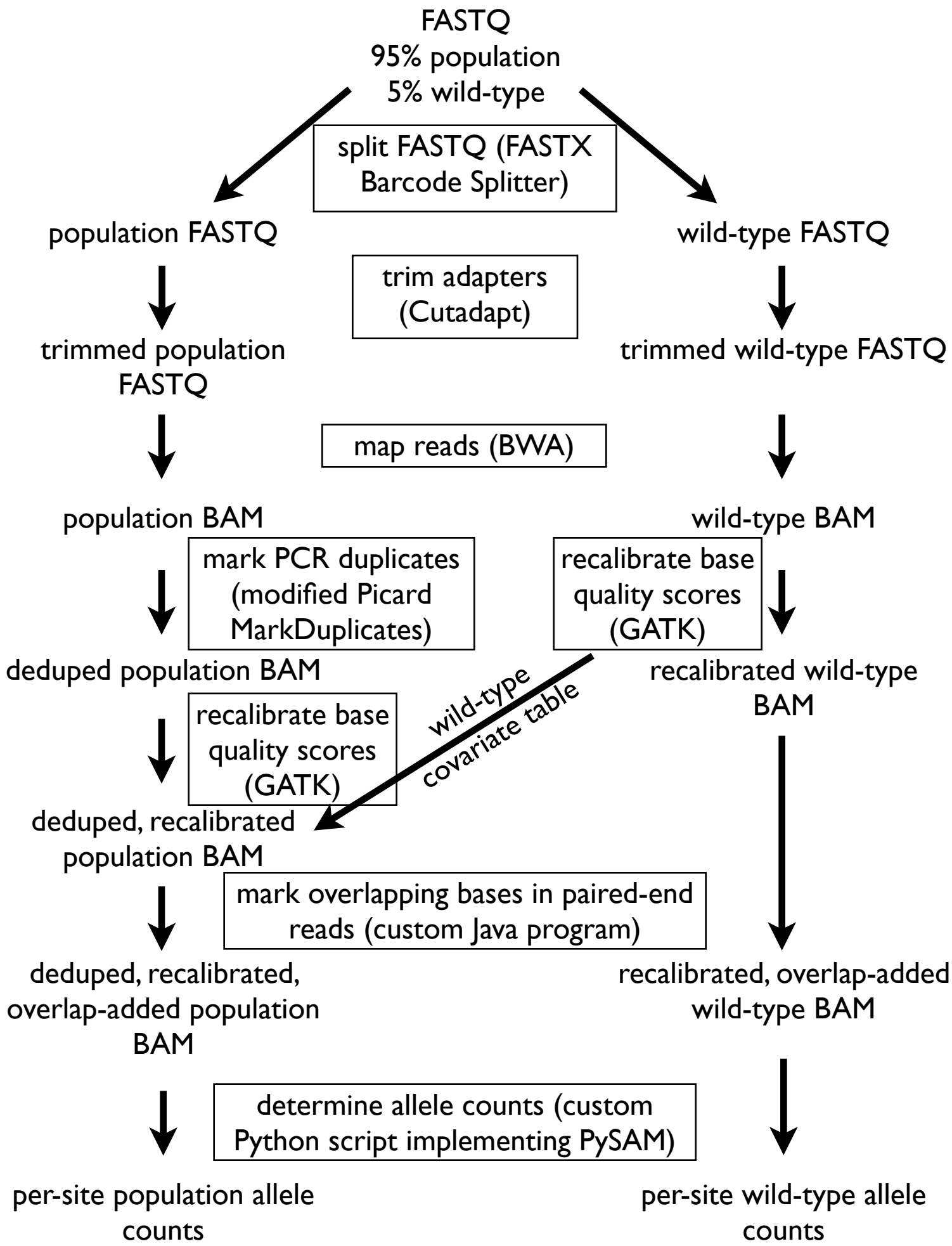

per-site population allele  
counts

per-site wild-type allele  
counts

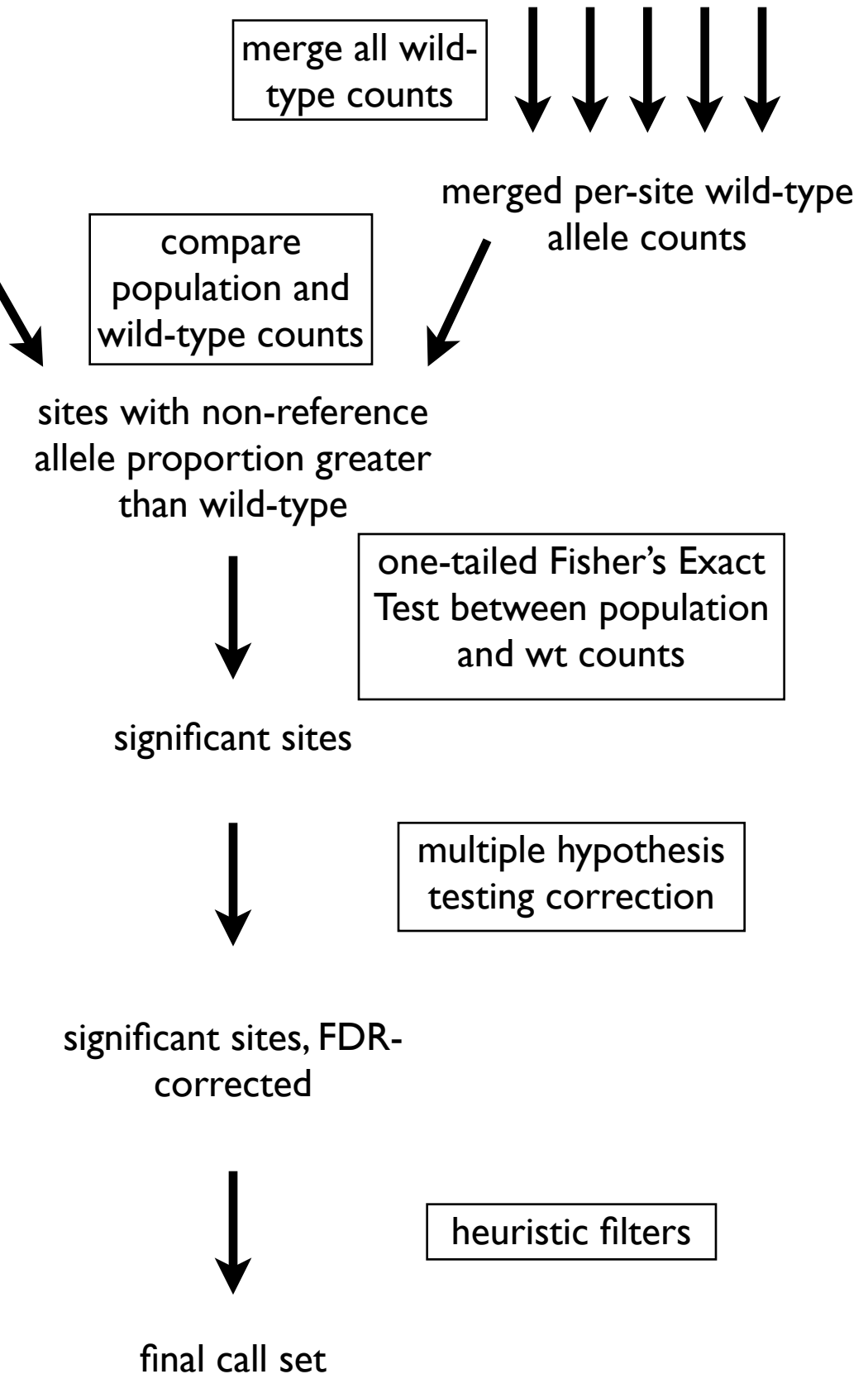

**Supplementary Figure 3.**

Supplement: Figure S3 — Diagram of the analysis pipeline used to call SNPs from population sequencing by converting raw data to allele counts and allele counts to SNP calls. Actions performed on data are in boxes, with programs used in parenthesis, if applicable. (PDF) [file pgen.1003972.s003.pdf]

**chr04:1014691**

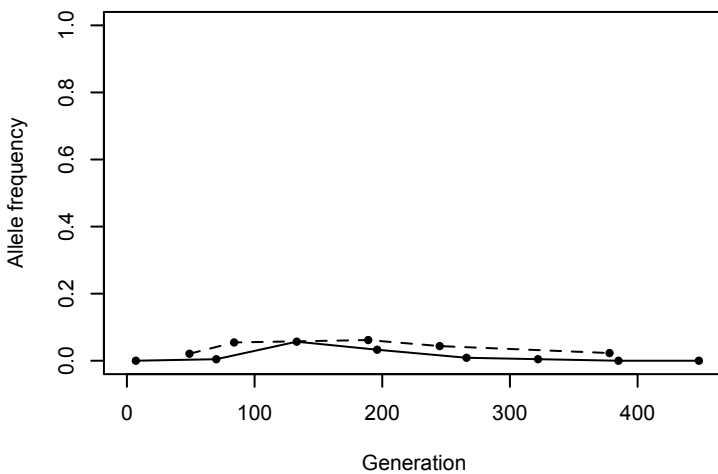

**chr02:521881**

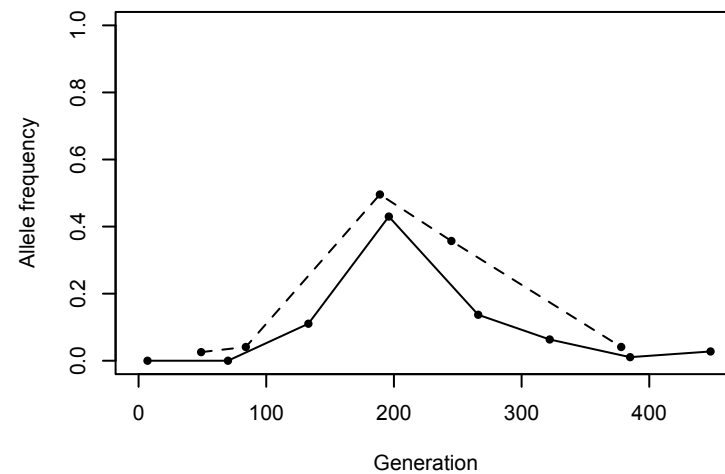

**chr11:64697**

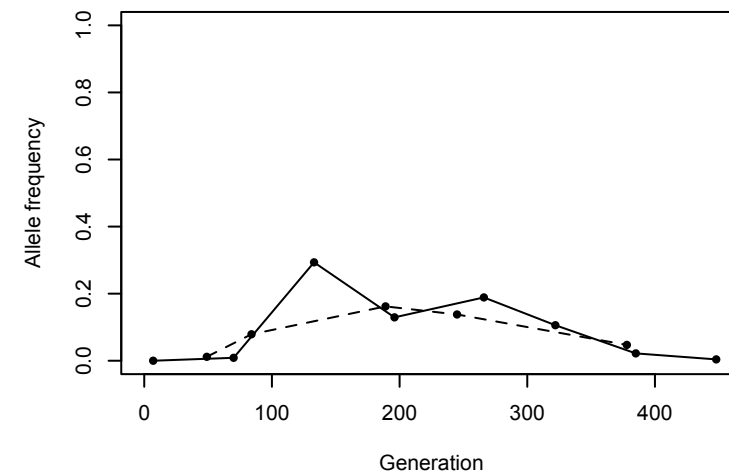

**chr15:893335**

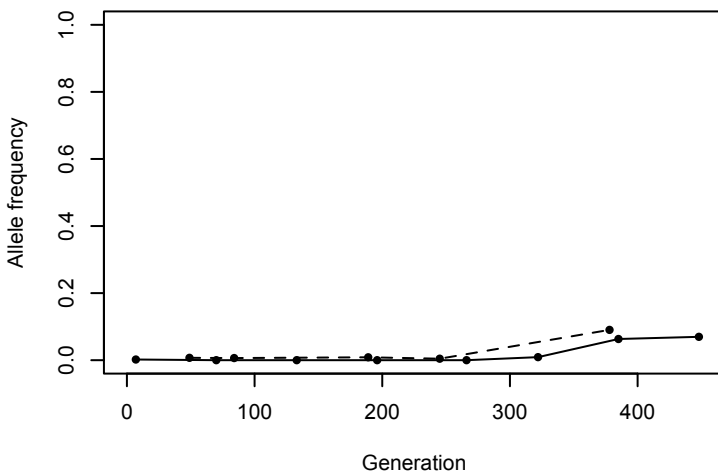

**chr16:422269**

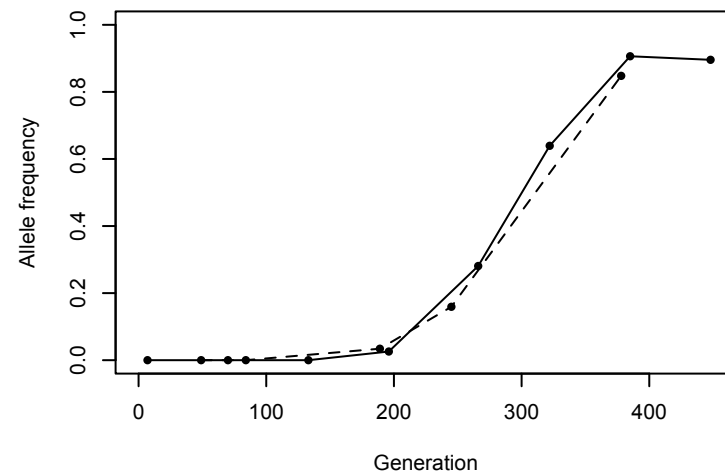

**chr04:1014981**

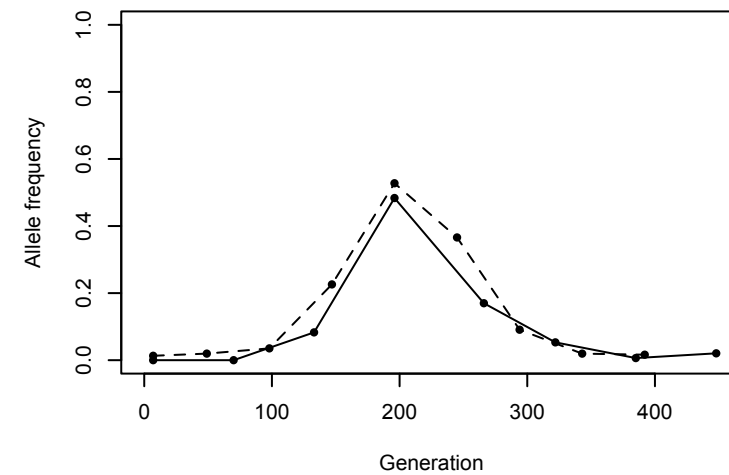

**Supplementary Figure 4.**

Supplement: Figure S4 — Validation of mutation allele frequency estimates using known mutations from E3. Solid lines are allele frequencies from this study. Dashed lines are frequencies of the same mutation as determined by either allele-specific quantitative PCR or quantitative sequencing. The time points that have data are different between the population sequencing and the validation, which contributes to the differences observed. (PDF) [file pgen.1003972.s004.pdf]

**A**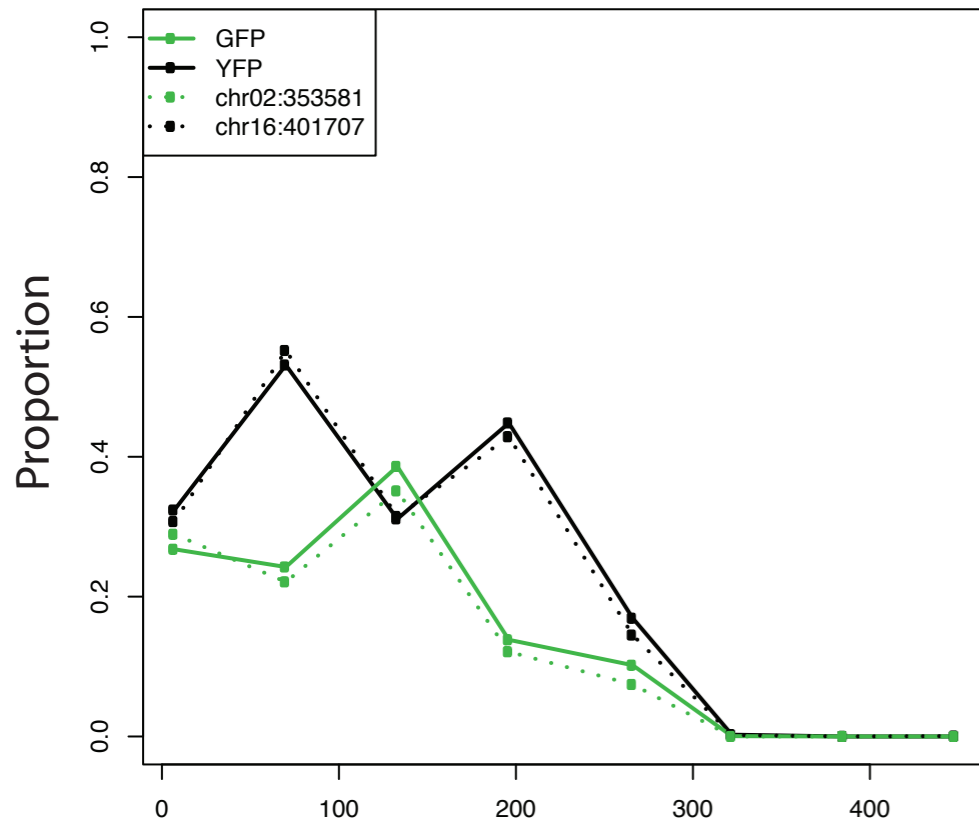**B**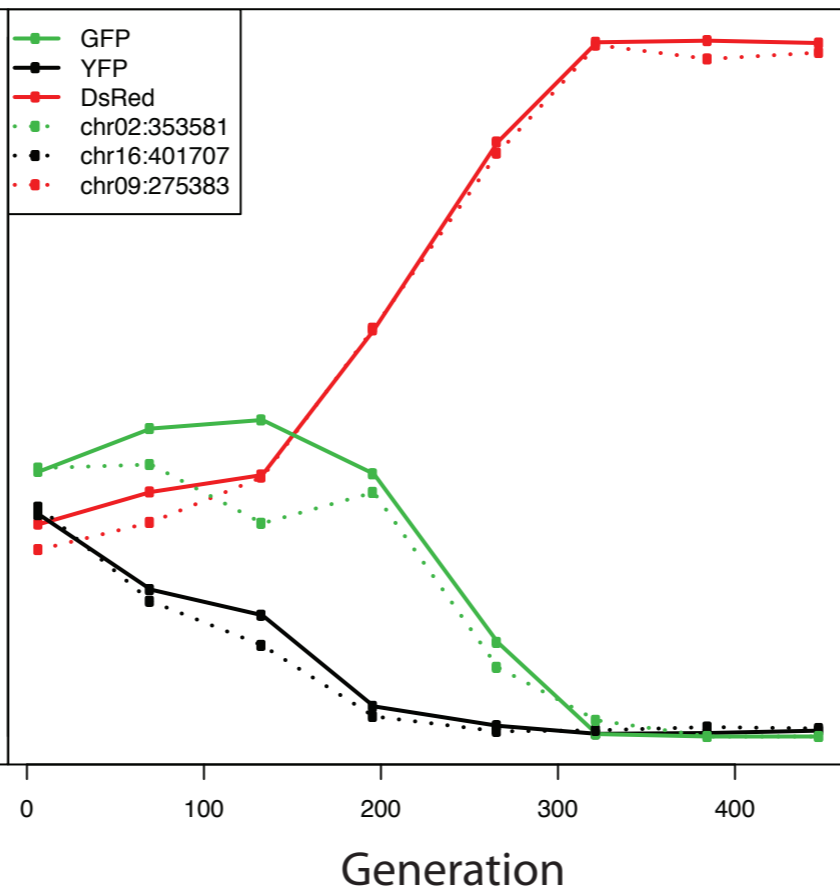**C**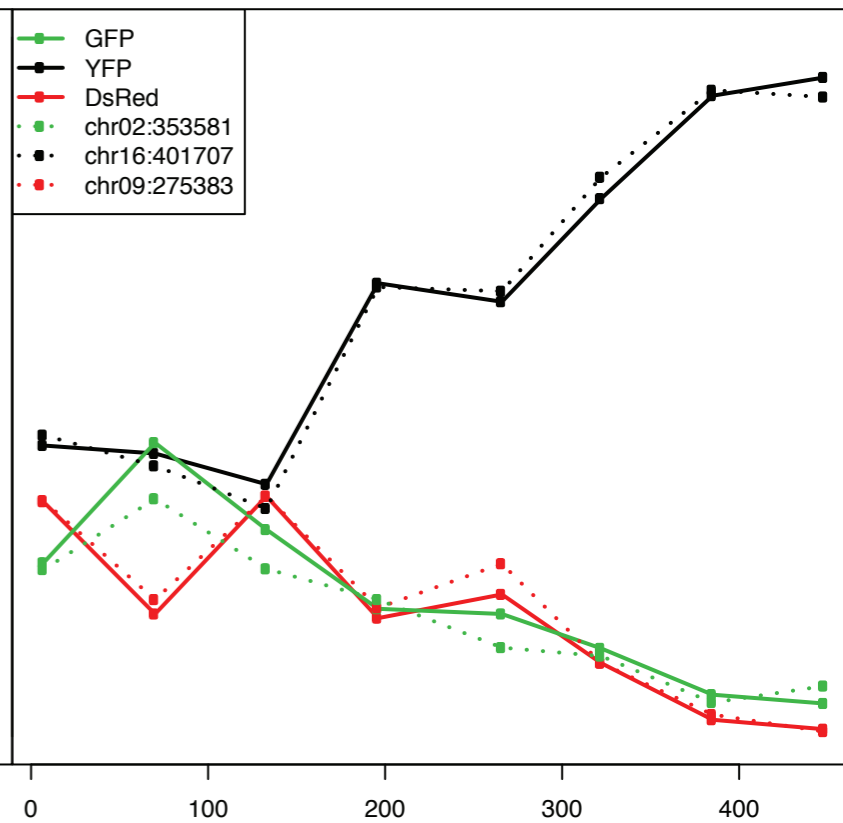**Supplementary Figure 5.**

Supplement: Figure S5 — Validation of mutation allele frequency estimates using known mutations carried by fluorescent reporter strains in A) E1, B) E2 and C) E3. Each strain carries a single SNP, except the DsRed strain used in E1. Solid lines indicate the proportions of each fluorescent protein reporter as determined by flow cytometry. Dotted lines show the allele frequency of the SNP carried by each fluorescent strain. (PDF) [file pgen.1003972.s005.pdf]
