# Supplementary material for: Whole Genome, Whole Population Sequencing Reveals That Loss of Signaling Networks Is the Major Adaptive Strategy in a Constant Environment
Source: PLoS Genet. 2013 Nov 21;9(11):e1003972. doi: 10.1371/journal.pgen.1003972 (PMC3836717; doi:10.1371/journal.pgen.1003972)
Supplement: Table S1 — Sequencing coverage of each sequencing library. Numbers are average fold sequencing coverage. The coverage from bases sequenced twice by overlapping read pairs are shown (OL), as well as bases sequence only once (non-OL). Only OL bases were used for the analysis to call SNPs. (PDF) [file pgen.1003972.s006.pdf]

Supplementary Table 1.

Generation	E1		E2		E3	
	OL	non-OL	OL	non-OL	OL	non-OL
7	594	60	266	22	562	76
70	791	118	312	27	516	74
133	867	111	288	23	459	59
196	832	118	302	26	516	111
266	902	152	358	41	610	78
322	821	120	371	44	531	94
385	1013	420	339	48	476	42
448	1046	447	452	88	637	103
