# Supplementary material for: Whole Genome, Whole Population Sequencing Reveals That Loss of Signaling Networks Is the Major Adaptive Strategy in a Constant Environment
Source: PLoS Genet. 2013 Nov 21;9(11):e1003972. doi: 10.1371/journal.pgen.1003972 (PMC3836717; doi:10.1371/journal.pgen.1003972)
Supplement: Table S2 — List of mutations discovered in each experiment. Once a mutation was discovered by the analysis pipeline, the frequency of the mutation was pulled from other time points to complete the allele frequency trajectory. (PDF) [file pgen.1003972.s007.pdf]

Table S2.

Mutations in E1

									Allele frequency at given generation							
Chromosome	Position	Ref Allele	Alt Allele	Gene(s)	Effect	Amino Acid Change	Codon Change	Codon Position	7	70	133	196	266	322	385	448
chr01	39261	G	A	GPB2	START_LOST	M/I	atG/atA	1	0	0.05243459	0.00385286	0.01013128	0	0	0	0
chr01	39425	A	G	GPB2	NON_SYNONYMOUS_CODING	Y/C	tAt/tGt	56	0.00286123	0	0.00088731	0.0019861	0	0	0.00313152	0.02065728
chr01	39834	G	A	GPB2	STOP_GAINED	W/*	tgG/tgA	192	0	0	0.00215916	0.10195227	0.02235031	0	0	0
chr03	235882	C	A	SED4	SYNONYMOUS_CODING	V/V	gtG/gtT	147	0	0	0	0	0	0	0.0018315	0.03259827
chr04	111908	T	C	SNF3	NON_SYNONYMOUS_CODING	L/S	tTa/tCa	110	0	0	0.02606178	0.15031983	0.03075397	0	0	0
chr04	112109	G	C	SNF3	NON_SYNONYMOUS_CODING	S/T	aGt/aCt	177	0	0.01041667	0.02896725	0.10597826	0.03001201	0	0	0
chr04	214167	T	C	RGT2	NON_SYNONYMOUS_CODING	Y/H	Tac/Cac	273	0	0	0.00787402	0.0270907	0.0020141	0	0	0
chr04	1014422	C	G	MTH1	NON_SYNONYMOUS_CODING	W/C	tgG/tgC	427	0	0	0	0.01152074	0.0780379	0.00111982	0	0
chr04	1014457	G	T	MTH1	NON_SYNONYMOUS_CODING	Q/K	Cag/Aag	416	0	0	0.00936768	0.0265252	0.00255428	0	0	0
chr04	1014707	A	C	MTH1	STOP_GAINED	Y/*	taT/taG	332	0	0.0089172	0.0154355	0.07751092	0.06924644	0.00321888	0	0
chr04	1014740	G	C	MTH1	NON_SYNONYMOUS_CODING	C/W	tgC/tgG	321	0	0	0.0031679	0.00220995	0.24282787	0.98303287	0.99913345	0.99823789
chr04	1014770	G	T	MTH1	STOP_GAINED	Y/*	taC/taA	311	0	0.00703235	0.1376038	0.00134048	0.01568154	0	0	0
chr04	1014968	A	T	MTH1	STOP_GAINED	Y/*	taT/taA	245	0	0.00158211	0.00254586	0.03146342	0.01408237	0	0	0
chr04	1014997	G	A	MTH1	STOP_GAINED	Q/*	Cag/Tag	236	0	0	0.0042735	0.02406015	0.0262069	0	0.00088968	0
chr04	1015236	A	C	MTH1	STOP_GAINED	L/*	tTa/tGa	156	0	0.01007326	0.02243313	0.01414677	0.01258851	0.0008881	0	0
chr04	1015386	G	T	MTH1	STOP_GAINED	S/*	tCa/tAa	106	0	0.00455581	0.03518124	0.02350176	0.01195219	0	0	0
chr09	400837	TACTGGCATCCATTTACTC	T	DAL81	CODON_DELETION	SIYSLAL/L	tccatttactcactggcatta/tta	357	0	0	0	0	0.035197	0.893491	0.986711	1
chrmt	28320	T	C	ATP6	UPSTREAM				0.00536089	0.00116357	0.00217971	0.00320859	0.00163258	0.00757479	0.0006589	0.00031782
chr06	70288	A	T	RIM15	STOP_GAINED	Y/*	taT/taA	1380	0	0	0	0.01660281	0.03787879	0.00121655	0	0
chr06	71386	G	C	RIM15	STOP_GAINED	Y/*	taC/taG	1014	0	0	0	0	0	0	0.00066181	0.03151674
chr10	428286	A	C	CYR1	NON_SYNONYMOUS_CODING	S/R	Agt/Cgt	1044	0	0.37113402	0.2	0.34660926	0.07276507	0.0022805	0	0
chr11	367181	A	G	RGT1	NON_SYNONYMOUS_CODING	Y/C	tAt/tGt	526	0	0	0.00271003	0.01768708	0.04111246	0	0.00078927	0
chr11	367297	G	C	RGT1	NON_SYNONYMOUS_CODING	V/L	Gtt/Ctt	565	0	0	0.00242131	0.02070646	0.08089097	0.00131234	0	0
chr11	367328	T	G	RGT1	NON_SYNONYMOUS_CODING	L/W	tTg/tGg	575	0	0	0	0.02751323	0.11285267	0.00115741	0	0
chr12	54429	T	C	VPS13	NON_SYNONYMOUS_CODING	I/V	Att/Gtt	3073	0	0	0.00114417	0.01816118	0.01256545	0	0	0
chr12	162823	TAA	TCAT	SSK1	FRAME_SHIFT				0	0	0	0	0.22638	0.986335	0.998209	0.996253
chr12	200286	C	T	ADE16	NON_SYNONYMOUS_CODING	G/R	Ggg/Agg	344	0	0	0	0	0.20884774	0.98847631	0.99826087	1
chr12	372230	G	A	HOG1	NON_SYNONYMOUS_CODING	G/D	gGt/gAt	204	0	0	0	0.01220505	0.07127273	0	0	0
chr12	372319	G	T	HOG1	STOP_GAINED	G/*	Gga/Tga	234	0	0	0	0.02328042	0.11365807	0.0010977	0	0
chr13	302056	TCACAGCAACATCGACAACGCAAGGTTTGACGAATTTGGAAGATTGCAAGATCTT	T	ERG5	CODON_DELETION			143	0	0	0	0	0.00361011	0.42857143	0.71428571	1
chr14	440309	C	A	RAS2	NON_SYNONYMOUS_CODING	V/F	Gtt/Ttt	88	0	0	0	0	0.02726473	0	0	0
chr14	684980	C	A	SSK2	STOP_GAINED	E/*	Gag/Tag	152	0	0	0.00324675	0.04701835	0.04468719	0.00109649	0	0
chr15	301909	G	A	HRD1	NON_SYNONYMOUS_CODING	P/L	cCt/cTt	376	0	0	0	0.00214753	0.02686023	0.00223359	1.61E-05	0
chr15	384462	T	A	CIN5,YOR029W	UPSTREAM,UPSTREAM				0	0.00120919	0.03067485	0.00119761	0	0	0	0
chr15	438512	A	C	YOR059C,Gene_tK(UUU)O	DOWNSTREAM,UPSTREAM				0	0	0	0	0.0049505	0.35714286	0.85714286	1
chr16	428459	A	G	VPS28	NON_SYNONYMOUS_CODING	H/R	cAt/cGt	178	0	0	0	0.00434153	0.05670103	0.00136054	0	0
chr16	522341	T	G	SWI1	NON_SYNONYMOUS_CODING	L/R	cTt/cGt	443	0	0	0	0.00104275	0.02341137	0	0	0

Mutations in E2

									Allele frequency at given generation							
Chromosome	Position	Ref Allele	Alt Allele	Gene(s)	Effect	Amino Acid Change	Codon Change	Codon Position	7	70	133	196	266	322	385	448
chr01	39261	G	A	GPB2	START_LOST	M/I	atG/atA	1	0	0	0.00294772	0.04086741	0.16442322	0.012442	0.01272351	0.01787002
chr03	184888	C	A	BPH1	NON_SYNONYMOUS_CODING	S/Y	tCt/tAt	1790	0	0	0	0.00766284	0.02208202	0.02578797	0.04	0.01456311
chr04	112987	G	T	SNF3	NON_SYNONYMOUS_CODING	V/F	Gtt/Ttt	470	0	0.00719425	0.03861004	0	0	0	0	0
chr04	214167	T	C	RGT2	NON_SYNONYMOUS_CODING	Y/H	Tac/Cac	273	0	0	0.00403226	0.09507042	0.06944444	0	0	0
chr04	1014763	T	G	MTH1	NON_SYNONYMOUS_CODING	N/H	Aat/Cat	314	0	0	0	0.04166667	0.19834711	0.03794038	0.03951368	0.0131579
chr04	1014850	T	A	MTH1	STOP_GAINED	R/*	Aga/Tga	285	0	0	0.00126597	0.29351251	0.42957695	0.6581447	0.67056437	0.79198941
chr04	1014958	C	A	MTH1	STOP_GAINED	E/*	Gag/Tag	249	0	0.00724638	0.03829787	0	0	0	0	0
chr04	1015139	A	T	MTH1	STOP_GAINED	C/*	tgT/tgA	188	0	0	0	0.03485255	0.00902935	0	0	0
chr04	1015401	G	T	MTH1	STOP_GAINED	S/*	tCa/tAa	101	0	0.01282051	0.04089219	0	0.00268817	0	0	0
chr04	1015447	T	A	MTH1	STOP_GAINED	K/*	Aaa/Taa	86	0	0	0	0.00355872	0.00925926	0.04892966	0.07166124	0.02320186
chr04	1393816	C	A	RMT2	SYNONYMOUS_CODING	G/G	ggG/ggT	253	0	0	0	0.0070922	0.01685393	0.00527705	0.08459215	0.0950324
chrmt	5237	AG	A		DELETION				0	0.080673	0.058112	0.52224	0.814544	0.892617	0.865747	0.903259
chrmt	48457	T	C	Gene_tS(UGA)Q2,VAR1	DOWNSTREAM,UPSTREAM				0	0.00034389	0.00103765	0.01205405	0.00651069	0.0004732	0	0
chr06	71813	A	C	RIM15	NON_SYNONYMOUS_CODING	M/R	aTg/aGg	872	0	0.14089347	0.07291667	0.33797909	0.10479042	0	0	0
chr07	31970	G	A	RMR1	NON_SYNONYMOUS_CODING	E/K	Gag/Aag	21	0	0.0027027	0.01829268	0.00808625	0.03217822	0.01474202	0.02956989	0.02227172
chr07	146779	T	C	CDC55	NON_SYNONYMOUS_CODING	D/G	gAc/gGc	204	0.00343643	0.09703504	0.0380117	0.53581662	0.81795511	0.98130841	0.97186701	0.99420849
chr08	368657	A	C	ECM14	NON_SYNONYMOUS_CODING	Y/D	Tac/Gac	380	0	0	0	0.004	0.00835655	0.00537634	0.04692082	0.07940447
chr10	178522	G	A	PBS2	NON_SYNONYMOUS_CODING	P/S	Cct/Tct	528	0	0	0.00322581	0.00952381	0.00464037	0.01160093	0.04057279	0.01086957
chr10	179074	C	A	PBS2	STOP_GAINED	G/*	Gga/Tga	344	0	0	0	0.00325733	0	0.015625	0.046875	0.10191083
chr10	619248	A	G	VPS25	NON_SYNONYMOUS_CODING	L/P	cTc/cCc	173	0	0	0.04545455	0.00302115	0.02941177	0.00715991	0.02898551	0.05150215
chr11	367541	G	T	RGT1	NON_SYNONYMOUS_CODING	C/F	tGt/tTt	646	0	0.00245098	0.00278552	0.01975309	0.02863436	0	0	0
chr11	368053	G	A	RGT1	NON_SYNONYMOUS_CODING	E/K	Gaa/Aaa	817	0	0	0	0.04271357	0.02702703	0.03151261	0.01818182	0.01570681
chr12	162282	C	A	SSK1	STOP_GAINED	E/*	Gaa/Taa	538	0	0	0	0	0	0	0.03188406	0.03929273
chr12	162304	A	T	SSK1	STOP_GAINED	Y/*	taT/taA	530	0	0	0	0	0	0.02272727	0.07925408	0.53454546
chr12	162807	G	A	SSK1	STOP_GAINED	Q/*	Caa/Taa	363	0	0	0	0	0.01044386	0.01358696	0.02064897	0.00809717
chr12	162975	G	A	SSK1	STOP_GAINED	Q/*	Cag/Tag	307	0	0	0	0	0.00208768	0.03207547	0.03981265	0.03448276
chr12	909689	C	T	COX8,CST9	DOWNSTREAM,DOWNSTREAM				0	0	0	0.00444444	0.0035461	0.03021148	0.01602564	0.00571429
chr13	302398	C	CGCCTAC	ERG5	CODON_INSERTION			30	0	0	0	0	0	0	0.00307692	0.11439114
chr14	680935	C	T	SSK2	STOP_GAINED	W/*	tGg/tAg	1500	0	0	0	0	0.00595238	0.02608696	0.02479339	0.01601831
chr14	681026	T	G	SSK2	NON_SYNONYMOUS_CODING	T/P	Act/Cct	1470	0	0	0	0	0.02475248	0.14761905	0.12142857	0.02819957
chr14	684767	G	A	SSK2	STOP_GAINED	Q/*	Cag/Tag	223	0	0	0	0	0.00232558	0.01455302	0.02110818	0.00606061
chr15	412148	G	T	WHI2	STOP_GAINED	E/*	Gaa/Taa	427	0	0	0	0	0.02040816	0.07692308	0.10361446	0.04789272
chr15	412274	C	T	WHI2	STOP_GAINED	R/*	Cga/Tga	469	0	0	0	0.00310059	0.00175224	0.06156304	0.08335231	0.01447027
chr15	591963	G	T	ARP8	NON_SYNONYMOUS_CODING	L/M	Ctg/Atg	209	0	0	0	0	0.00547945	0.03225807	0.01829268	0.00803213
chr16	581589	T	A	Gene_tK(CUU)P,RPA135	UPSTREAM,UPSTREAM				0	0	0	0.01195219	0.03952569	0.07142857	0.11009174	0.0915493

Mutations in E3

									Allele frequency at given generation							
Chromosome	Position	Ref Allele	Alt Allele	Gene(s)	Effect	Amino Acid Change	Codon Change	Codon Position	7	70	133	196	266	322	385	448
chr01	39485	C	G	GPB2	STOP_GAINED	S/*	tCa/tGa	76	0	0	0	0	0	0	0.00385699	0.05954187
chr01	173820	T	C	CDC15	NON_SYNONYMOUS_CODING	Y/C	tAt/tGt	439	0	0	0	0	0	0	0.00531915	0.09627728
chr01	191184	A	G	YAT1	NON_SYNONYMOUS_CODING	Q/R	cAa/cGa	331	0	0	0	0	0.00817439	0.09937888	0.14285714	0.01876676
chr02	521881	C	T	IRA1	NON_SYNONYMOUS_CODING	R/K	aGa/aAa	1583	0	0	0.11036036	0.4298893	0.13703704	0.06336634	0.0106383	0.02760736
chr04	629878	G	A	UBC13	START_LOST	M/I	atG/atA	1	0	0	0	0.002457	0.00225225	0.02962963	0.01511335	0.07515658
chr04	1014583	G	A	MTH1	STOP_GAINED	Q/*	Cag/Tag	374	0	0.00375235	0.02439024	0.01426025	0	0	0	0
chr04	1014691	G	A	MTH1	STOP_GAINED	Q/*	Caa/Taa	338	0	0.00451807	0.05676127	0.0328125	0.00882724	0.00459418	0	0
chr04	1014981	A	T	MTH1	STOP_GAINED	L/*	tTa/tAa	241	0	0	0.0828877	0.4835443	0.16985138	0.0531401	0.00632911	0.0204461
chr04	1015077	G	T	MTH1	STOP_GAINED	S/*	tCg/tAg	209	0	0	0	0.05145414	0.09839357	0.03874092	0	0
chr05	433605	G	A	GLC7	NON_SYNONYMOUS_CODING	D/N	Gac/Aac	196	0	0	0	0	0	0	0	0.03803487
chr06	70566	C	A	RIM15	STOP_GAINED	E/*	Gag/Tag	1288	0	0.00573614	0.03766478	0.00480769	0	0	0	0
chr06	70913	C	T	RIM15	STOP_GAINED	W/*	tGg/tAg	1172	0	0	0	0.04799253	0.11369602	0.05194288	-0.0008873	-0.0008873
chr06	71961	T	C	RIM15	NON_SYNONYMOUS_CODING	K/E	Aag/Gag	823	0	0	0	0	0	0.05486726	0.05427975	0.01445087
chr06	73706	C	A	RIM15	NON_SYNONYMOUS_CODING	C/F	tGc/tTc	241	0	0	0.0015748	0.00888889	0.01716247	0.00280112	0	0
chr07	146522	C	G	CDC55	NON_SYNONYMOUS_CODING	E/Q	Gaa/Caa	290	0	0	0	0	0	0	0	0.04761905
chr07	408768	C	T	TIF4632	SYNONYMOUS_CODING	A/A	gcG/gcA	279	0	0	0	0	0	0	0.01490066	0.04405875
chr08	244533	T	A	OSH3	NON_SYNONYMOUS_CODING	V/E	gTa/gAa	651	0	0	0	0	0.00494234	0.03676471	0.01333333	0.00478469
chr08	245024	G	T	OSH3	STOP_GAINED	E/*	Gag/Tag	815	0	0	0	0	0	0	0.0016835	0.02997602
chr09	401526	G	A	DAL81	NON_SYNONYMOUS_CODING	A/T	Gca/Aca	584	0	0	0	0	0.02014389	0.17793594	0.43462247	0.65189048
chr10	158444	C	T	LCB3	NON_SYNONYMOUS_CODING	S/F	tCc/tTc	86	0	0	0	0	0.00209644	0	0.00518135	0.02363636
chr10	159332	T	G	LCB3	NON_SYNONYMOUS_CODING	L/R	cTa/cGa	382	0	0	0	0	0.00524476	0.03802281	0.0190678	0.00163934
chr10	250988	G	A	BCK1	NON_SYNONYMOUS_CODING	G/D	gGt/gAt	1245	0	0	0	0	0	0	0.00743494	0.10895884
chr10	517521	C	T	Gene_tM(CAU)J3,POL32	DOWNSTREAM,UPSTREAM				0	0	0	0	0	0	0.01265823	0.0455312
chr10	619870	G	T	VPS25	UPSTREAM				0	0	0	0	0	0	0.00170068	0.13788487
chr10	640231	C	T	YJR115W	NON_SYNONYMOUS_CODING	A/V	gCc/gTc	97	0	0	0	0.00673401	0.01714286	0.03287197	0.02356902	0.05675676
chr11	64697	T	C	MNN4	NON_SYNONYMOUS_CODING	K/E	Aag/Gag	924	0	0.0087146	0.29336735	0.12933025	0.18874773	0.10588235	0.02189781	0.00399202
chr11	434707	A	G	BYE1	SYNONYMOUS_CODING	T/T	acT/acC	57	0	0	0	0.01410935	0.05349183	0.28870968	0.47931035	0.70533333
chr12	404866	G	T	ACE2	NON_SYNONYMOUS_CODING	H/N	Cac/Aac	653	0	0	0	0	0	0	0.00387597	0.1016731
chr12	404904	C	A	ACE2	NON_SYNONYMOUS_CODING	C/F	tGc/tTc	640	0	0	0	0	0.00506329	0.00898204	0	0.01875733
chr12	404904	C	T	ACE2	NON_SYNONYMOUS_CODING	C/Y	tGc/tAc	640	0	0	0	0.00203542	0.00429759	0.01420436	0.01193272	0.04729995
chr12	406567	CA	C	ACE2	FRAMESHIFT			85	0	0	0	0	0	0	0	0.04203
chr12	662933	C	G	GSY2,HSP60	DOWNSTREAM,DOWNSTREAM				0	0	0	0	0	0	0.01535088	0.05504587
chr12	697147	T	G	YSH1,DBP9	DOWNSTREAM,UPSTREAM				0	0	0.00332226	0.02881844	0.06823529	0.00914634	0.00660066	0
chr14	482049	G	T	APJ1	NON_SYNONYMOUS_CODING	C/F	tGt/tTt	220	0	0	0	0.00511945	0.03048781	0.01565217	0.00174216	0.00144092
chr15	174575	T	G	IRA2	NON_SYNONYMOUS_CODING	M/R	aTg/aGg	1169	0	0	0	0	0	0	0.00468384	0.10186757
chr15	176389	A	T	IRA2	NON_SYNONYMOUS_CODING	I/F	Att/Ttt	1774	0	0	0	0	0.01105845	0.04307116	0.02439024	0.00438597
chr15	893335	C	A	SLY41	NON_SYNONYMOUS_CODING	W/L	tGg/tTg	253	0	0	0	0	0	0.00907441	0.06318083	0.06965944
chr15	1013378	C	G	PDE2	NON_SYNONYMOUS_CODING	G/R	Ggt/Cgt	482	0	0	0	0	0	0	0.00879765	0.05752212
chr15	1013813	C	A	PDE2	STOP_GAINED	E/*	Gaa/Taa	337	0	0	0	0	0.01013514	0.15612648	0.20855615	0.03622047
chr15	1036410	G	C	NDD1	STOP_GAINED	S/*	tCa/tGa	21	0	0	0	0	0	0.00281294	0.00651466	0.04563233
chr16	79761	T	G	GAL4	NON_SYNONYMOUS_CODING	N/H	Aac/Cac	866	0	0	0.00299401	0.03278689	0.1375	0.0359116	0	0
chr16	422269	C	A	MUK1	STOP_GAINED	S/*	tCa/tAa	441	0	0	0	0.0258216	0.28080808	0.63925729	0.90625	0.89566929
chr16	615985	C	T	ATH1	STOP_GAINED	Q/*	Caa/Taa	203	0	0	0	0	0	0	0	0.02718676
chr16	663257	A	G	ATG11	NON_SYNONYMOUS_CODING	W/R	Tgg/Cgg	473	0	0	0	0	0	0	0.00319295	0.03740391
chr16	745437	G	A	YPR109W	NON_SYNONYMOUS_CODING	R/K	aGa/aAa	250	0	0	0	0	0	0	0.00346621	0.06116643
