# Supplementary material for: Whole Genome, Whole Population Sequencing Reveals That Loss of Signaling Networks Is the Major Adaptive Strategy in a Constant Environment
Source: PLoS Genet. 2013 Nov 21;9(11):e1003972. doi: 10.1371/journal.pgen.1003972 (PMC3836717; doi:10.1371/journal.pgen.1003972)
Supplement: Table S3 — Gene Ontology (GO) biological process enrichments for each experiment. All genes hit with at least one mutation were the input for the GO enrichment analysis, and only GO terms with an FDR-corrected p-value of 0.01 or less are shown. (PDF) [file pgen.1003972.s008.pdf]

Supplementary Table 3.

E1

GOID	GO_term	Cluster frequency	Background frequency	P-value	FDR	Expected FP	Gene(s) annotated to the term
7165	signal transduction	9 out of 23 genes, 39.1%	224 out of 6359 background genes, 3.5%	8.32E-06	0	0	*GPB2,RGT2,SNF3,MTH1,CYR1,SSK1,HOG1,RAS2,SSK2*
23052	signaling	9 out of 23 genes, 39.1%	230 out of 6359 background genes, 3.6%	1.04E-05	0	0	*GPB2,RGT2,SNF3,MTH1,CYR1,SSK1,HOG1,RAS2,SSK2*
7154	cell communication	10 out of 23 genes, 43.5%	329 out of 6359 background genes, 5.2%	1.65E-05	0	0	*GPB2,RGT2,SNF3,MTH1,DAL81,CYR1,SSK1,HOG1,RAS2,SSK2*
50794	regulation of cellular process	15 out of 23 genes, 65.2%	1090 out of 6359 background genes, 17.1%	8.10E-05	0	0	*GPB2,SED4,RGT2,SNF3,MTH1,RIM15,DAL81,CYR1,RGT1,SSK1,HOG1,RAS2,SSK2,CIN5,SWI1*
50789	regulation of biological process	15 out of 23 genes, 65.2%	1125 out of 6359 background genes, 17.7%	0.00012	0	0	*GPB2,SED4,RGT2,SNF3,MTH1,RIM15,DAL81,CYR1,RGT1,SSK1,HOG1,RAS2,SSK2,CIN5,SWI1*
65007	biological regulation	16 out of 23 genes, 69.6%	1322 out of 6359 background genes, 20.8%	0.00014	0	0	*GPB2,SED4,RGT2,SNF3,MTH1,RIM15,DAL81,CYR1,RGT1,VPS13,SSK1,HOG1,RAS2,SSK2,CIN5,SWI1*
15758	glucose transport	3 out of 23 genes, 13.0%	10 out of 6359 background genes, 0.2%	0.00107	0	0	*SNF3,MTH1,RGT1*
34284	response to monosaccharide stimulus	3 out of 23 genes, 13.0%	10 out of 6359 background genes, 0.2%	0.00107	0	0	*GPB2,RGT2,SNF3*
9746	response to hexose stimulus	3 out of 23 genes, 13.0%	10 out of 6359 background genes, 0.2%	0.00107	0	0	*GPB2,RGT2,SNF3*
9749	response to glucose stimulus	3 out of 23 genes, 13.0%	10 out of 6359 background genes, 0.2%	0.00107	0	0	*GPB2,RGT2,SNF3*
9743	response to carbohydrate stimulus	3 out of 23 genes, 13.0%	11 out of 6359 background genes, 0.2%	0.00147	0	0	*GPB2,RGT2,SNF3*
42221	response to chemical stimulus	8 out of 23 genes, 34.8%	352 out of 6359 background genes, 5.5%	0.00422	0	0	*GPB2,RGT2,SNF3,RIM15,DAL81,HOG1,RAS2,CIN5*
50896	response to stimulus	12 out of 23 genes, 52.2%	925 out of 6359 background genes, 14.5%	0.00528	0	0.02	*GPB2,RGT2,SNF3,MTH1,RIM15,DAL81,CYR1,SSK1,HOG1,RAS2,SSK2,CIN5*
51716	cellular response to stimulus	11 out of 23 genes, 47.8%	766 out of 6359 background genes, 12.0%	0.00545	0	0.02	*GPB2,RGT2,SNF3,MTH1,RIM15,DAL81,CYR1,SSK1,HOG1,RAS2,SSK2*
7231	osmosensory signaling pathway	3 out of 23 genes, 13.0%	17 out of 6359 background genes, 0.3%	0.00598	0	0.02	*SSK1,HOG1,SSK2*
34287	detection of monosaccharide stimulus	2 out of 23 genes, 8.7%	3 out of 6359 background genes, 0.0%	0.00824	0	0.02	*RGT2,SNF3*
51594	detection of glucose	2 out of 23 genes, 8.7%	3 out of 6359 background genes, 0.0%	0.00824	0	0.02	*RGT2,SNF3*
51606	detection of stimulus	2 out of 23 genes, 8.7%	3 out of 6359 background genes, 0.0%	0.00824	0	0.02	*RGT2,SNF3*
9593	detection of chemical stimulus	2 out of 23 genes, 8.7%	3 out of 6359 background genes, 0.0%	0.00824	0	0.02	*RGT2,SNF3*
9730	detection of carbohydrate stimulus	2 out of 23 genes, 8.7%	3 out of 6359 background genes, 0.0%	0.00824	0	0.02	*RGT2,SNF3*
9732	detection of hexose stimulus	2 out of 23 genes, 8.7%	3 out of 6359 background genes, 0.0%	0.00824	0	0.02	*RGT2,SNF3*

E2

GOID	GO_term	Cluster frequency	Background frequency	P-value	FDR	Expected FP	Gene(s) annotated to the term
7165	signal transduction	8 out of 24 genes, 33.3%	224 out of 6359 background genes, 3.5%	0.00018	0	0	*GPB2,RGT2,SNF3,AFR1,MTH1,PBS2,SSK1,SSK2*
23052	signaling	8 out of 24 genes, 33.3%	230 out of 6359 background genes, 3.6%	0.00022	0	0	*GPB2,RGT2,SNF3,AFR1,MTH1,PBS2,SSK1,SSK2*
7154	cell communication	9 out of 24 genes, 37.5%	329 out of 6359 background genes, 5.2%	0.00029	0	0	*GPB2,RGT2,SNF3,AFR1,MTH1,PBS2,VPS25,SSK1,SSK2*
15749	monosaccharide transport	4 out of 24 genes, 16.7%	24 out of 6359 background genes, 0.4%	0.0003	0	0	*SNF3,MTH1,HXT6,RGT1*
8645	hexose transport	4 out of 24 genes, 16.7%	24 out of 6359 background genes, 0.4%	0.0003	0	0	*SNF3,MTH1,HXT6,RGT1*
15758	glucose transport	3 out of 24 genes, 12.5%	10 out of 6359 background genes, 0.2%	0.00105	0	0	*SNF3,MTH1,RGT1*
34284	response to monosaccharide stimulus	3 out of 24 genes, 12.5%	10 out of 6359 background genes, 0.2%	0.00105	0	0	*GPB2,RGT2,SNF3*
9746	response to hexose stimulus	3 out of 24 genes, 12.5%	10 out of 6359 background genes, 0.2%	0.00105	0	0	*GPB2,RGT2,SNF3*
9749	response to glucose stimulus	3 out of 24 genes, 12.5%	10 out of 6359 background genes, 0.2%	0.00105	0	0	*GPB2,RGT2,SNF3*
50896	response to stimulus	13 out of 24 genes, 54.2%	925 out of 6359 background genes, 14.5%	0.00119	0	0	*GPB2,BPH1,RGT2,SNF3,AFR1,MTH1,RIM15,PBS2,VPS25,SSK1,SSK2,WHI2,ARP8*
9743	response to carbohydrate stimulus	3 out of 24 genes, 12.5%	11 out of 6359 background genes, 0.2%	0.00144	0	0	*GPB2,RGT2,SNF3*
8643	carbohydrate transport	4 out of 24 genes, 16.7%	41 out of 6359 background genes, 0.6%	0.00271	0	0	*SNF3,MTH1,HXT6,RGT1*
7231	osmosensory signaling pathway	3 out of 24 genes, 12.5%	17 out of 6359 background genes, 0.3%	0.00583	0	0	*PBS2,SSK1,SSK2*
51716	cellular response to stimulus	11 out of 24 genes, 45.8%	766 out of 6359 background genes, 12.0%	0.00767	0	0	*GPB2,RGT2,SNF3,AFR1,MTH1,RIM15,PBS2,VPS25,SSK1,SSK2,ARP8*
34287	detection of monosaccharide stimulus	2 out of 24 genes, 8.3%	3 out of 6359 background genes, 0.0%	0.00768	0	0.02	*RGT2,SNF3*
51594	detection of glucose	2 out of 24 genes, 8.3%	3 out of 6359 background genes, 0.0%	0.00768	0	0.02	*RGT2,SNF3*
51606	detection of stimulus	2 out of 24 genes, 8.3%	3 out of 6359 background genes, 0.0%	0.00768	0	0.02	*RGT2,SNF3*
9593	detection of chemical stimulus	2 out of 24 genes, 8.3%	3 out of 6359 background genes, 0.0%	0.00768	0	0.02	*RGT2,SNF3*
9730	detection of carbohydrate stimulus	2 out of 24 genes, 8.3%	3 out of 6359 background genes, 0.0%	0.00768	0	0.02	*RGT2,SNF3*
9732	detection of hexose stimulus	2 out of 24 genes, 8.3%	3 out of 6359 background genes, 0.0%	0.00768	0	0.02	*RGT2,SNF3*

E3

GOID	GO_term	Cluster frequency	Background frequency	P-value	FDR	Expected FP	Gene(s) annotated to the term
50789	regulation of biological process	20 out of 37 genes, 54.1%	1125 out of 6359 background genes, 17.7%	0.00019	0	0	*GPB2,CDC15,IRA1,UBC13,MTH1,GLC7,RIM15,CDC55,OSH3,DAL81,BCK1,LCB3,VPS25,BYE1,ACE2,HSP60,IRA2,PDE2,NDD1,GAL4*
50794	regulation of cellular process	19 out of 37 genes, 51.4%	1090 out of 6359 background genes, 17.1%	0.00062	0	0	*GPB2,CDC15,IRA1,UBC13,MTH1,GLC7,RIM15,CDC55,OSH3,DAL81,BCK1,LCB3,VPS25,BYE1,ACE2,IRA2,PDE2,NDD1,GAL4*
65007	biological regulation	20 out of 37 genes, 54.1%	1322 out of 6359 background genes, 20.8%	0.00266	0.01	0.02	*GPB2,CDC15,IRA1,UBC13,MTH1,GLC7,RIM15,CDC55,OSH3,DAL81,BCK1,LCB3,VPS25,BYE1,ACE2,HSP60,IRA2,PDE2,NDD1,GAL4*
46580	negative regulation of Ras protein signal transduction	3 out of 37 genes, 8.1%	8 out of 6359 background genes, 0.1%	0.00327	0.01	0.02	*GPB2,IRA1,IRA2*
51058	negative regulation of small GTPase mediated signal transduction	3 out of 37 genes, 8.1%	8 out of 6359 background genes, 0.1%	0.00327	0	0.02	*GPB2,IRA1,IRA2*
7154	cell communication	10 out of 37 genes, 27.0%	329 out of 6359 background genes, 5.2%	0.00392	0	0.02	*GPB2,IRA1,MTH1,DAL81,BCK1,LCB3,VPS25,IRA2,PDE2,GAL4*
50896	response to stimulus	16 out of 37 genes, 43.2%	925 out of 6359 background genes, 14.5%	0.00735	0	0.02	*GPB2,IRA1,UBC13,MTH1,GLC7,RIM15,DAL81,BCK1,LCB3,POL32,VPS25,MNN4,IRA2,PDE2,GAL4,ATH1*
