# Supplementary material for: Whole Genome, Whole Population Sequencing Reveals That Loss of Signaling Networks Is the Major Adaptive Strategy in a Constant Environment
Source: PLoS Genet. 2013 Nov 21;9(11):e1003972. doi: 10.1371/journal.pgen.1003972 (PMC3836717; doi:10.1371/journal.pgen.1003972)
Supplement: Table S4 — GO terms were organized into networks and overlap between GO networks was calculated to assess the functional reproducibility of adaptation between experiments (see methods). GO terms in bold/italic/underline are terms that define a network, and that are shared between all three experiments. GO terms in italic/underline define the network and are shared only between E1 and E2. (PDF) [file pgen.1003972.s009.pdf]

Supplementary Table 4.

**E1:** 2/3 networks shared with all, 3/3 networks shared with E2							
**Networks 1 & 2**							
65007	biological regulation	15 out of 19	1339 out of 5	8.59E-05	0	0	GPB2/YAL056W:SED4/YCR067C:RGT2/YDL138W:SNF3/YDL194W:MTH1/YDR277C:RIM15/YFL033C:DAL81/YIR023W:CYR1/YJL005W:RGT1/YKL038W:VPS13/YLL040C:SSK1/YLR006C:HOG1/YLR113W:RAS2/YNL098C:SSK2/YNR031C:SWI1/YPL016W
50789	***regulation of biological process***	14 out of 19	1145 out of 5	0.00012	0	0	GPB2/YAL056W:SED4/YCR067C:RGT2/YDL138W:SNF3/YDL194W:MTH1/YDR277C:RIM15/YFL033C:DAL81/YIR023W:CYR1/YJL005W:RGT1/YKL038W:SSK1/YLR006C:HOG1/YLR113W:RAS2/YNL098C:SSK2/YNR031C:SWI1/YPL016W
50794	regulation of cellular process	14 out of 19	1101 out of 5	7.20E-05	0	0	GPB2/YAL056W:SED4/YCR067C:RGT2/YDL138W:SNF3/YDL194W:MTH1/YDR277C:RIM15/YFL033C:DAL81/YIR023W:CYR1/YJL005W:RGT1/YKL038W:SSK1/YLR006C:HOG1/YLR113W:RAS2/YNL098C:SSK2/YNR031C:SWI1/YPL016W
7165	signal transduction	9 out of 19 g	221 out of 58	2.13E-06	0	0	GPB2/YAL056W:RGT2/YDL138W:SNF3/YDL194W:MTH1/YDR277C:CYR1/YJL005W:SSK1/YLR006C:HOG1/YLR113W:RAS2/YNL098C:SSK2/YNR031C
7231	osmosensory signaling pathway	3 out of 19 g	17 out of 582	0.0045	0	0	SSK1/YLR006C:HOG1/YLR113W:SSK2/YNR031C
51716	cellular response to stimulus	12 out of 19	775 out of 58	0.00014	0	0	GPB2/YAL056W:RGT2/YDL138W:SNF3/YDL194W:MTH1/YDR277C:RIM15/YFL033C:DAL81/YIR023W:CYR1/YJL005W:SSK1/YLR006C:HOG1/YLR113W:RAS2/YNL098C:SSK2/YNR031C:SWI1/YPL016W
23052	signaling	9 out of 19 g	227 out of 58	2.70E-06	0	0	GPB2/YAL056W:RGT2/YDL138W:SNF3/YDL194W:MTH1/YDR277C:CYR1/YJL005W:SSK1/YLR006C:HOG1/YLR113W:RAS2/YNL098C:SSK2/YNR031C
7165	signal transduction	9 out of 19 g	221 out of 58	2.13E-06	0	0	GPB2/YAL056W:RGT2/YDL138W:SNF3/YDL194W:MTH1/YDR277C:CYR1/YJL005W:SSK1/YLR006C:HOG1/YLR113W:RAS2/YNL098C:SSK2/YNR031C
7154	***cell communication***	11 out of 19	338 out of 58	2.47E-07	0	0	GPB2/YAL056W:RGT2/YDL138W:SNF3/YDL194W:MTH1/YDR277C:DAL81/YIR023W:CYR1/YJL005W:SSK1/YLR006C:HOG1/YLR113W:RAS2/YNL098C:SSK2/YNR031C:SWI1/YPL016W
**Network 3**							
34284	response to monosaccharide stimulus	3 out of 19 g	10 out of 582	0.00081	0	0	GPB2/YAL056W:RGT2/YDL138W:SNF3/YDL194W
9746	response to hexose stimulus	3 out of 19 g	10 out of 582	0.00081	0	0	GPB2/YAL056W:RGT2/YDL138W:SNF3/YDL194W
9749	response to glucose stimulus	3 out of 19 g	10 out of 582	0.00081	0	0	GPB2/YAL056W:RGT2/YDL138W:SNF3/YDL194W
50896	***response to stimulus***	12 out of 19	932 out of 58	0.00103	0	0	GPB2/YAL056W:RGT2/YDL138W:SNF3/YDL194W:MTH1/YDR277C:RIM15/YFL033C:DAL81/YIR023W:CYR1/YJL005W:SSK1/YLR006C:HOG1/YLR113W:RAS2/YNL098C:SSK2/YNR031C:SWI1/YPL016W
9743	response to carbohydrate stimulus	3 out of 19 g	11 out of 582	0.00111	0	0	GPB2/YAL056W:RGT2/YDL138W:SNF3/YDL194W
42221	response to chemical stimulus	8 out of 19 g	354 out of 58	0.00167	0	0	GPB2/YAL056W:RGT2/YDL138W:SNF3/YDL194W:RIM15/YFL033C:DAL81/YIR023W:HOG1/YLR113W:RAS2/YNL098C:SWI1/YPL016W
34287	detection of monosaccharide stimulus	2 out of 19 g	3 out of 5822	0.00698	0	0	RGT2/YDL138W:SNF3/YDL194W
51594	detection of glucose	2 out of 19 g	3 out of 5822	0.00698	0	0	RGT2/YDL138W:SNF3/YDL194W
51606	detection of stimulus	2 out of 19 g	3 out of 5822	0.00698	0	0	RGT2/YDL138W:SNF3/YDL194W
9593	detection of chemical stimulus	2 out of 19 g	3 out of 5822	0.00698	0	0	RGT2/YDL138W:SNF3/YDL194W
9730	detection of carbohydrate stimulus	2 out of 19 g	3 out of 5822	0.00698	0	0	RGT2/YDL138W:SNF3/YDL194W
9732	detection of hexose stimulus	2 out of 19 g	3 out of 5822	0.00698	0	0	RGT2/YDL138W:SNF3/YDL194W
**Network 4**							
15758	*glucose transport*	3 out of 19 g	10 out of 582	0.00081	0	0	SNF3/YDL194W:MTH1/YDR277C:RGT1/YKL038W
**E2:** 3/4 networks shared with all, 4/4 networks shared with E1							
**Network 1**							
50789	***regulation of biological process***	12 out of 18	1145 out of 5	0.00353	0	0.04	GPB2/YAL056W:RGT2/YDL138W:SNF3/YDL194W:MTH1/YDR277C:RIM15/YFL033C:CDC55/YGL190C:PBS2/YJL128C:VPS25/YJR102C:RGT1/YKL038W:SSK1/YLR006C:SSK2/YNR031C:WHI2/YOR043W
**Network 2**							
7154	***cell communication***	8 out of 18 g	338 out of 58	0.00061	0	0	GPB2/YAL056W:RGT2/YDL138W:SNF3/YDL194W:MTH1/YDR277C:PBS2/YJL128C:VPS25/YJR102C:SSK1/YLR006C:SSK2/YNR031C
23052	signaling	7 out of 18 g	227 out of 58	0.00054	0	0	GPB2/YAL056W:RGT2/YDL138W:SNF3/YDL194W:MTH1/YDR277C:PBS2/YJL128C:SSK1/YLR006C:SSK2/YNR031C
7165	signal transduction	7 out of 18 g	221 out of 58	0.00045	0	0	GPB2/YAL056W:RGT2/YDL138W:SNF3/YDL194W:MTH1/YDR277C:PBS2/YJL128C:SSK1/YLR006C:SSK2/YNR031C
7231	osmosensory signaling pathway	3 out of 18 g	17 out of 582	0.0032	0	0.04	PBS2/YJL128C:SSK1/YLR006C:SSK2/YNR031C
**Network 3**							
50896	***response to stimulus***	12 out of 18	932 out of 58	0.00038	0	0	GPB2/YAL056W:BPH1/YCR032W:RGT2/YDL138W:SNF3/YDL194W:MTH1/YDR277C:RIM15/YFL033C:PBS2/YJL128C:VPS25/YJR102C:SSK1/YLR006C:SSK2/YNR031C:WHI2/YOR043W:ARP8/YOR141C
51716	cellular response to stimulus	10 out of 18	775 out of 58	0.00513	0.01	0.06	GPB2/YAL056W:RGT2/YDL138W:SNF3/YDL194W:MTH1/YDR277C:RIM15/YFL033C:PBS2/YJL128C:VPS25/YJR102C:SSK1/YLR006C:SSK2/YNR031C:ARP8/YOR141C
9628	response to abiotic stimulus	5 out of 18 g	126 out of 58	0.00584	0.01	0.12	BPH1/YCR032W:PBS2/YJL128C:SSK1/YLR006C:SSK2/YNR031C:WHI2/YOR043W
34284	response to monosaccharide stimulus	3 out of 18 g	10 out of 582	0.00057	0	0	GPB2/YAL056W:RGT2/YDL138W:SNF3/YDL194W
9746	response to hexose stimulus	3 out of 18 g	10 out of 582	0.00057	0	0	GPB2/YAL056W:RGT2/YDL138W:SNF3/YDL194W
9749	response to glucose stimulus	3 out of 18 g	10 out of 582	0.00057	0	0	GPB2/YAL056W:RGT2/YDL138W:SNF3/YDL194W
9743	response to carbohydrate stimulus	3 out of 18 g	11 out of 582	0.00079	0	0	GPB2/YAL056W:RGT2/YDL138W:SNF3/YDL194W
34287	detection of monosaccharide stimulus	2 out of 18 g	3 out of 5822	0.00527	0.01	0.1	RGT2/YDL138W:SNF3/YDL194W
51594	detection of glucose	2 out of 18 g	3 out of 5822	0.00527	0.01	0.1	RGT2/YDL138W:SNF3/YDL194W
51606	detection of stimulus	2 out of 18 g	3 out of 5822	0.00527	0.01	0.1	RGT2/YDL138W:SNF3/YDL194W
9593	detection of chemical stimulus	2 out of 18 g	3 out of 5822	0.00527	0.01	0.1	RGT2/YDL138W:SNF3/YDL194W
9730	detection of carbohydrate stimulus	2 out of 18 g	3 out of 5822	0.00527	0.01	0.1	RGT2/YDL138W:SNF3/YDL194W
9732	detection of hexose stimulus	2 out of 18 g	3 out of 5822	0.00527	0.01	0.1	RGT2/YDL138W:SNF3/YDL194W
**Network 4**							
15758	*glucose transport*	3 out of 18 g	10 out of 582	0.00057	0	0	SNF3/YDL194W:MTH1/YDR277C:RGT1/YKL038W
15749	monosaccharide transport	3 out of 18 g	24 out of 582	0.00941	0.01	0.16	SNF3/YDL194W:MTH1/YDR277C:RGT1/YKL038W
8645	hexose transport	3 out of 18 g	24 out of 582	0.00941	0.01	0.16	SNF3/YDL194W:MTH1/YDR277C:RGT1/YKL038W
**E3:** 3/6 networks shared with all							
**Network 1**							
65007	biological regulation	18 out of 28	1339 out of 5	0.00096	0	0	GPB2/YAL056W:CDC15/YAR019C:IRA1/YBR140C:UBC13/YDR092W:MTH1/YDR277C:GLC7/YER133W:RIM15/YFL033C:CDC55/YGL190C:OSH3/YHR073W:DAL81/YIR023W:BCK1/YJL095W:LCB3/YJL134W:BYE1/YKL005C:ACE2/YLR131C:IRA2/YOL081W:PDE2/YOR360C:NDD1/YOR372C:GAL4/YPL248C
50789	***regulation of biological process***	18 out of 28	1145 out of 5	8.32E-05	0	0	GPB2/YAL056W:CDC15/YAR019C:IRA1/YBR140C:UBC13/YDR092W:MTH1/YDR277C:GLC7/YER133W:RIM15/YFL033C:CDC55/YGL190C:OSH3/YHR073W:DAL81/YIR023W:BCK1/YJL095W:LCB3/YJL134W:BYE1/YKL005C:ACE2/YLR131C:IRA2/YOL081W:PDE2/YOR360C:NDD1/YOR372C:GAL4/YPL248C
50794	regulation of cellular process	18 out of 28	1101 out of 5	4.45E-05	0	0	GPB2/YAL056W:CDC15/YAR019C:IRA1/YBR140C:UBC13/YDR092W:MTH1/YDR277C:GLC7/YER133W:RIM15/YFL033C:CDC55/YGL190C:OSH3/YHR073W:DAL81/YIR023W:BCK1/YJL095W:LCB3/YJL134W:BYE1/YKL005C:ACE2/YLR131C:IRA2/YOL081W:PDE2/YOR360C:NDD1/YOR372C:GAL4/YPL248C
**Network 2**							
7154	***cell communication***	9 out of 28 g	338 out of 58	0.00482	0	0	GPB2/YAL056W:IRA1/YBR140C:MTH1/YDR277C:DAL81/YIR023W:BCK1/YJL095W:LCB3/YJL134W:IRA2/YOL081W:PDE2/YOR360C:GAL4/YPL248C
**Network 3**							
50896	***response to stimulus***	14 out of 28	932 out of 58	0.00804	0	0	GPB2/YAL056W:IRA1/YBR140C:UBC13/YDR092W:MTH1/YDR277C:GLC7/YER133W:RIM15/YFL033C:DAL81/YIR023W:BCK1/YJL095W:LCB3/YJL134W:MNN4/YKL201C:IRA2/YOL081W:PDE2/YOR360C:GAL4/YPL248C:ATH1/YPR026W
**Network 5**							
46580	negative regulation of Ras protein signal transduction	3 out of 28 g	8 out of 5822	0.00152	0	0	GPB2/YAL056W:IRA1/YBR140C:IRA2/YOL081W
51058	negative regulation of small GTPase mediated signal transduction	3 out of 28 g	8 out of 5822	0.00152	0	0	GPB2/YAL056W:IRA1/YBR140C:IRA2/YOL081W
**Network 6**							
35556	intracellular signal transduction	6 out of 28 g	127 out of 58	0.00674	0	0	GPB2/YAL056W:IRA1/YBR140C:BCK1/YJL095W:LCB3/YJL134W:IRA2/YOL081W:PDE2/YOR360C
**Network 7**							
1900372	negative regulation of purine nucleotide biosynthetic process	2 out of 28 g	2 out of 5822	0.00618	0	0	IRA1/YBR140C:IRA2/YOL081W
1900543	negative regulation of purine nucleotide metabolic process	2 out of 28 g	2 out of 5822	0.00618	0	0	IRA1/YBR140C:IRA2/YOL081W
30800	negative regulation of cyclic nucleotide metabolic process	2 out of 28 g	2 out of 5822	0.00618	0	0	IRA1/YBR140C:IRA2/YOL081W
30803	negative regulation of cyclic nucleotide biosynthetic process	2 out of 28 g	2 out of 5822	0.00618	0	0	IRA1/YBR140C:IRA2/YOL081W
30809	negative regulation of nucleotide biosynthetic process	2 out of 28 g	2 out of 5822	0.00618	0	0	IRA1/YBR140C:IRA2/YOL081W
30815	negative regulation of cAMP metabolic process	2 out of 28 g	2 out of 5822	0.00618	0	0	IRA1/YBR140C:IRA2/YOL081W
30818	negative regulation of cAMP biosynthetic process	2 out of 28 g	2 out of 5822	0.00618	0	0	IRA1/YBR140C:IRA2/YOL081W
45980	negative regulation of nucleotide metabolic process	2 out of 28 g	2 out of 5822	0.00618	0	0	IRA1/YBR140C:IRA2/YOL081W
